# A Mesh Updating Framework for Shell-Element Finite Element Models Based on Projection of Anomaly Regions Extracted from Point-Cloud Data

**DOI:** 10.3390/s26185950

**Published:** 2026-09-20

**Authors:** Jiexiu Wang, Mayuko Nishio

**Affiliations:** 1 Department of Engineering Mechanics and Energy, University of Tsukuba, Tsukuba 305-8573, Japan; 2 Institute of Systems and Information Engineering, University of Tsukuba, Tsukuba 305-8573, Japan; nishio@kz.tsukuba.ac.jp

**Keywords:** steel structures, point cloud data, finite element model, shell element, anomaly detection, re-meshing

## Abstract

**Highlights:**

**What are the main findings?**

- A modular framework maps surface anomalies onto a shell-element FE model driven by computer-vision methods using point-cloud data.
- Across the evaluated anomaly regions, IoU ranged from 0.44 to 0.87, while predicted capacity reduction differed from manual updating by 1.2 percentage points.

**What are the implications of the main findings?**

- Combining point-cloud geometry and color information enables direct damage mapping onto the corresponding shell-element FE model, avoiding 2D–3D data matching.
- The workflow provides a basis for preliminary FEA-based assessment of thin-walled steel structures.

**Abstract:**

Finite element (FE) analysis that incorporates structural damages extracted using computer vision (CV) methods based on three-dimensional point-cloud data (PCD) is effective for evaluating the residual capacity of structures. In this study, observable surface damages are regarded as surface anomaly regions on the structure. Based on this assumption, a modular mesh-model updating framework is proposed to incorporate damage information into shell-element FE models through the anomaly detection from point-cloud data. The framework comprises three modules: a perception module for the anomaly detection and anomaly-region localization, a description module for the anomaly-region quantification, and a remeshing module for mapping the anomaly region onto the FE model. Its performance was evaluated using a steel angle member specimen with artificially introduced anomaly regions representing damage. The updating results were evaluated in terms of both geometric accuracy and FE analysis (FEA) applicability by comparison with the reference FE model manually constructed from the designed anomaly-region geometries. The Intersection over Union (IoU) between corresponding anomaly regions in the updated and reference models ranges from 0.44 to 0.87, including the individual plate results for cross-surface cases. Through static elastoplastic analysis, the predicted reductions in ultimate load-bearing capacity differed by approximately 1.2 percentage points between the two updated models. Moreover, local stress redistribution showed qualitative agreement, although quantitative discrepancies remained in the magnitudes and locations of stress concentrations. Together with several supporting components, the proposed framework provides an effective workflow for integrating PCD-based CV techniques into FE model updating and has potential for FEA-based damage assessment of structures.

## 1. Introduction

Evaluating the performances of civil structures under damaged conditions is critical owing to their crucial role in supporting societal infrastructures. Numerical analyses, such as finite element analysis (FEA), are commonly used for this purpose. Typically, an FEA-based evaluation requires a two-step process. The first step involves collecting structural data, including design and inspection data. The second step involves an analysis that incorporates a representation of all simplified information [1]. The accuracy of the FEA-based evaluation of structural performance depends not only on the accuracy of the boundary and load modeling but also on the fidelity of the damage representations within the finite element (FE) model. Although theoretical and empirical studies on boundary conditions and loading are well-established, unified standards for the representations of detected damage are still lacking. Although numerous damage detection methods have been adopted, including visual inspection, non-destructive testing, and structural health monitoring, these conservative methods are difficult to implement, subjective, or limited to providing intuitive data, thereby facing challenges in efficiently quantifying the structural damages.

To address these issues, computer-vision (CV) techniques, which offer the advantages of remote, non-contact data capture and robust, automated data processing, have been introduced for the damage assessment. In essence, CV techniques mimic human visual perception of the environment, enabling them to replace visual inspection. With the rapid development of computer hardware and state-of-the-art machine learning algorithms, CV techniques have become one of the most effective approaches for quantifying the structural damages.

In this paper, we provide a comprehensive exposition of the current status of research on CV-based damage assessment. Based on the principle outlined in a previous study [2], common observable structural damages are classified into three types: crack-like (1D), surface (2D), and volumetric (3D). Crack-like damage refers to narrow but through-thickness cracks in steel plates. Surface damage, such as steel corrosion, also called surficial or textural damages in some studies, is often assessed based on surface distribution. Volumetric damage includes wide concrete cracks, spalling, deep corrosion, steel deformation (dents, bulges, and buckling), and component failure, which are typically accompanied by measurable volumetric changes. Computer vision is primarily used to extract and quantify the location and geometric features of those damage regions. The following reviews the related studies conducted to date.

Notably, image-based methods, which represent the most extensively studied category in CV research, are now capable of detecting and quantifying nearly all types of damage. Classical image processing algorithms include the threshold algorithm [3], edge algorithm [4], and region-growing algorithm [5]. Selecting the most appropriate algorithm for various detection tasks, integrating machine learning with multiple detection systems to achieve efficiency and automation, and establishing a virtual dataset of infrastructure using a generative adversarial network have become crucial aspects of CV-based damage assessment [6]. For example, Xu et al. [7] used direct measurements of the bounding box framing concrete cracks, concrete spalling, and rebar exposure to quantify these phenomena. Qi et al. [8] proposed a three-step CV-based framework to automatically identify the length, maximum width, and area of concrete cracks visible in images. However, to measure the three-dimensional volume of damage, images must be combined using a depth camera to obtain RGB-D information. Beckman et al. [9] proposed an RGB-D-based method that fits a plane to calculate damage volume using a depth channel. This approach can be considered an application of point-cloud data (PCD).

Recent advances in sensor technologies have facilitated the acquisition of 3D PCD of structures, enabling the development of PCD-based CV methods. Mainstream approaches, which leverage the geometric information of point clouds, either detect abnormal regions by identifying abrupt geometric changes between adjacent points or segment damages by comparing the point cloud with a CAD model or an undamaged reference. These methods have been extensively applied to solid model structures. Given the relative maturity of research on image-based damage detection, earlier studies predominantly identified damage through image processing. The depth information of the detected damage can be obtained by registering or matching images with PCD. Building on this concept, Yuan et al. [10] proposed a 3D crack quantification method that employs a deep learning algorithm to detect cracks from a single image and project them onto the corresponding region on a 3D point cloud reconstructed using a stereo camera. Chen et al. [11] proposed an improved Otsu-based fusion method for integrating 3D point clouds and 2D images to detect and segment concrete cracks. This method demonstrated high robustness to lighting and texture noise, achieving an F1 score of 86.7%. Kong et al. [12] established a vision-aided framework for the quantification of concrete spalling damage. The framework integrates image-based damage segmentation and depth matching using a perspective transformation-based registration method. These studies all leverage the high resolution of images and the depth information of PCD. However, the accuracy of the projection and the computational cost associated with the registration process remain difficult to control.

With improvements in the resolution of 3D reconstruction and the advancement of point-cloud processing algorithms, researchers have begun to explore direct damage detection using PCD. Zhang et al. [13] used boundary fitting and surface deviation based on point clouds to quantify spalling damages by estimating the volume of spalled material. To validate the proposed methods at the analysis level, several studies have conducted analytical tests on FE models updated using PCD. Zhang et al. [14] proposed an iterative closest point (ICP)-based DLT registration framework to integrate crack information captured by 2D images into 3D point-cloud models for automated FE model generation. They validated the proposed method through a nonlinear analysis of a cracked reinforced concrete beam, achieving good computational stability. Song et al. [15] directly utilized a point-cloud model of a concrete wall containing cracks to extract the crack points and map them onto a solid FE model. The elements containing the crack points were deactivated using the birth-death element to update the model. Subsequently, nonlinear FEA was performed to validate the updated model. However, these studies primarily address damage assessment and FE model updating for concrete structures. The damage characterizes significant geometric changes which are very easy to identify and quantify in point clouds.

In recent years, the number of studies on point-cloud-based damage assessment methods for thin-walled structures has been gradually increasing. Lopes et al. [16] calibrated an FE model of a deformed steel storage tank using 3D laser scanning data to reflect the actual shell geometry. The calibrated model was used for nonlinear static, modal, and dynamic buckling analyses. The results confirmed that point-cloud-calibrated FE models enable a more realistic evaluation of the buckling behavior of damaged steel structures. Sangani and Agarwal [17] developed a comprehensive framework for evaluating the residual capacity of dented steel tubular columns. They constructed computationally efficient FE meshes using the surface geometries of damaged specimens captured by dense point clouds. The FE-based residual strength estimations exhibited good agreement with the experimental results, demonstrating the suitability of point-cloud-based geometric reconstruction for realistic damage simulations. Wang et al. [18] proposed a 3D laser scanning-based intelligent safety assessment framework for existing steel structures. The framework integrates YOLOv5-based feature detection and Poisson surface reconstruction for geometric modeling, followed by FEA. This method combines 2D deep learning detection on point-cloud projections with 3D geometric reconstruction, enabling accurate identification of holes and direct FE model generation for safety evaluation. These studies primarily focus on reconstructing geometric defects, such as deformation, dents, and holes, from point-cloud data and incorporating the reconstructed geometry into FE models to evaluate their effects on structural performance.

Corrosion is one of the most prevalent forms of damage in steel structures, and consequently, the need for FEA of corroded steel structures is increasing. It must be simplified to align with different types of elements. Rúa et al. [19] applied a PCD-based method that enables the efficient modeling of corroded steel profiles using 1D beam elements for subsequent FE model simulations. Tzortzinis et al. [20,21] conducted a series of studies on corroded beams from a decommissioned bridge to validate a PCD-based method for reconstructing a shell-element FE model with corrosion deterioration. These studies focus on representing corrosion-induced material loss in FE models of steel members. The damage representations are based on the remaining geometry or cross-sectional properties, enabling assessment of the influence of corrosion on structural response and residual capacity. However, when corrosion is treated as surface damage and the focus is solely on the area and distribution of corrosion, the target of detection is to identify, segment, and quantify the damaged surface regions. For these tasks, the RGB and intensity information in the point cloud can also be used to detect damage. Aijazi et al. [22] proposed a PCD-based method for automatically detecting and quantifying the corrosion on large ship hulls by separating the illumination-invariant color component from the HSV values of the points. Hou et al. [23] compared the efficiency and accuracy of four data-clustering algorithms based on both the RGB and intensity information of PCD in detecting three types of textural damage, including corrosion. Ono et al. [24] developed an automatic method for detecting metallic corrosion by utilizing both 3D shapes and intensities of a LiDAR point cloud and achieved high robustness. These studies demonstrate the use of point-cloud color or intensity information to detect and quantify surface damage. Their primary focus is identifying damage regions and characterizing their spatial distribution and extent.

In summary, the current research on CV-based structural damage assessment methods primarily focuses on modeling (based on point clouds) and damage identification, classification, and localization (based on images or point clouds). Studies on damage quantification methods are also increasing; however, most studies have been limited to measuring damage dimensions. Moreover, there are far more studies related to mass solid structures than to shell structures. Research on incorporating the damage information quantified by PCD-based CV methods in numerical models remains relatively underdeveloped, as does the evaluation of the modeling method through FE analysis.

Specifically, current studies exhibit the following three significant limitations:

(1) Most existing studies utilize only geometric information embedded in PCD to either complement the missing depth information in images or compute geometric deviations by comparing the scanned model with an undamaged reference. Consequently, these approaches are limited to surface damage in concrete or large deformations in thin-walled structures, where geometric changes are clearly observable.

(2) Very few studies have exploited texture information contained in PCD. This information can provide sufficient accuracy for identifying surface damage on structures, thereby eliminating the need for a cumbersome matching process between 2D images and 3D point clouds. However, most of these studies have focused primarily on damage detection.

(3) In terms of model updating, common shell-element FE models of thin-walled structures and their corresponding surface point clouds exhibit geometric inconsistencies, which require special handling during registration and mapping. Nevertheless, research in this area remains limited.

To address these issues, this study proposes a modular framework for incorporating surface anomaly regions extracted from PCD into existing shell-element FE models of thin-walled steel structures. Its specific methodological contributions are as follows:

(1) The framework jointly utilizes the color and geometric information contained in PCD and adapts and integrates established algorithms for registration, anomaly detection, boundary extraction, and local remeshing, while accounting for the characteristics of thin-walled structures, their FE models, and point-cloud data. This enables surface anomaly identification, geometric boundary representation, and mesh-model updating to be integrated within a unified workflow. The modular organization allows individual components to be refined independently.

(2) The registration and mapping procedures account for the geometric relationship between physical plate surfaces and shell mid-planes, allowing anomaly boundaries extracted from the surface PCD to be located on the corresponding planes of the existing shell-element mesh.

(3) A plane-based segmentation strategy handles anomaly regions extending across multiple planes. Boundary feature points are matched along common joint lines to preserve boundary consistency and mesh continuity during local remeshing.

The framework is evaluated using one steel angle member specimen with ten predefined, high-contrast anomaly regions through comparisons with a manually updated reference FE model in terms of geometric accuracy and FE responses.

The objective of this study is to propose a PCD-based framework for updating the mesh of shell-element FE models of steel structures with anomaly regions. The remainder of this paper is organized as follows. Section 2 presents the methodology based on the dataset by discussing the remeshing procedures, focusing on the key algorithms employed and their corresponding optimization techniques proposed. Section 3 details the anomaly region configurations and FEA setup used in this study and evaluates the updated model through comparisons with a manually constructed reference model in terms of both geometric accuracy and consistency of the numerical FE response. Finally, Section 4 concludes the study by summarizing the results and evaluating the applicability of the proposed approach. In this study, “damage” and “damaged” refer to structural deterioration, including actual damage discussed in the literature and prescribed thickness loss introduced in the FE models. “Anomaly” and “Anomalous” refer to an artificially introduced color-anomaly region and its geometric representation during point-cloud processing and mesh-model updating. These regions serve as geometric symbols for surface damage. The thickness loss used in the FE comparison is prescribed rather than inferred from the color information.

## 2. Methodology

This section presents the mesh updating framework for shell-element FE models with surface structural damage information from 3D point-cloud data. The key algorithms are demonstrated using a simulated specimen of the steel angle member with anomaly regions denoting surface damage.

### 2.1. Overview of Proposed Framework

**Figure 1:**
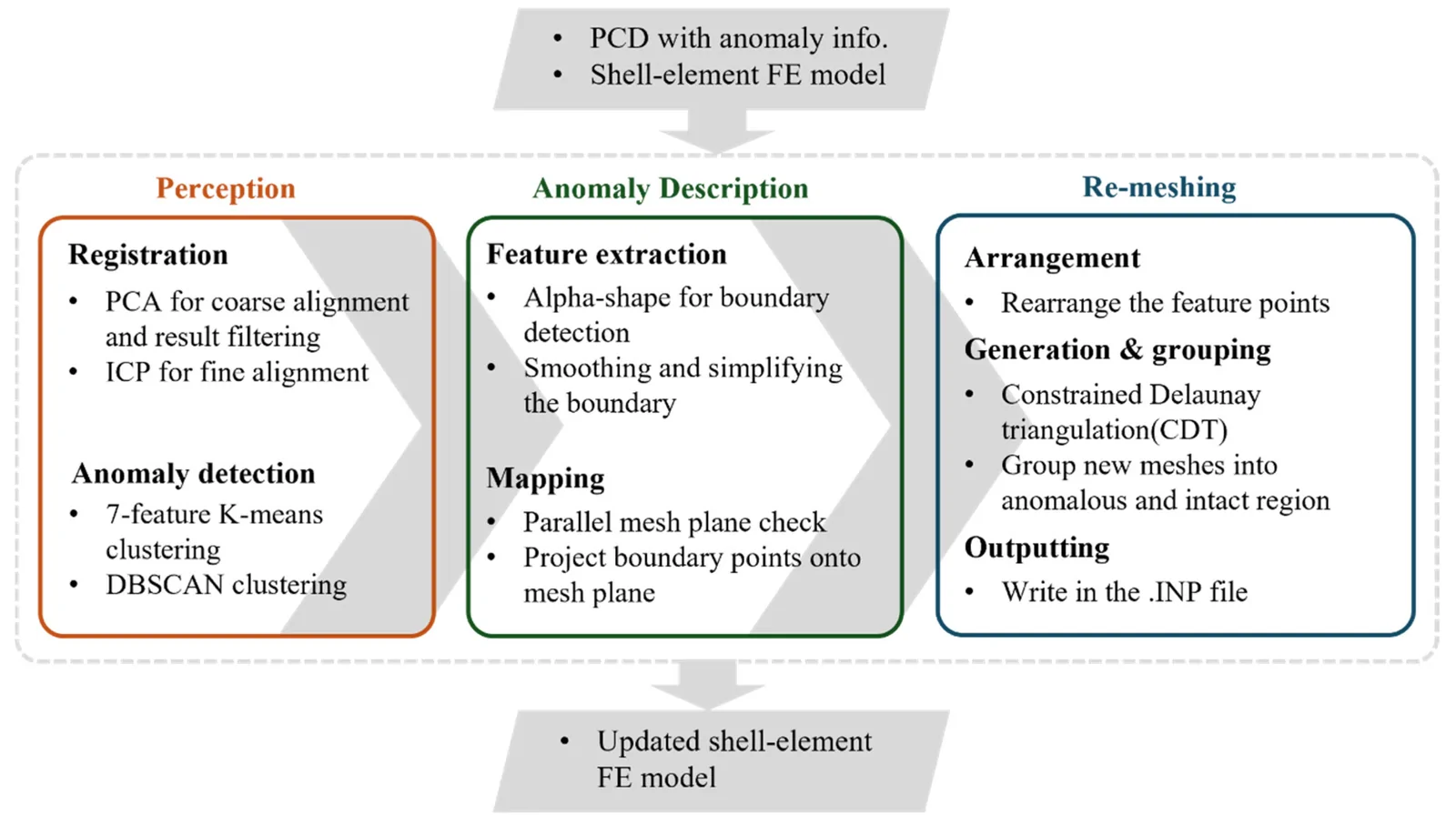
PCD-based CV-driven mesh updating framework for shell-element FE model.

Figure 1 presents an overview of the proposed framework, which comprises three modules: perception, anomaly description, and remeshing. The perception module is used to extract and locate anomalous points from PCD. It comprises two components: registration and anomaly detection. This study focuses on surface damage on thin-walled structures, e.g., corrosions on steel structures. The proposed framework is based on the assumption that observable surface damage can be characterized as surface anomaly regions. Thus, the anomaly detection is the process to extract points in anomaly regions based on color information.

The anomaly description module is used to represent and map anomaly regions by featuring extracted point sets. This module comprises two components: feature extraction, and mapping. The feature extraction component is for extracting features that represent the anomalous point set based on the boundary detection. The mapping component reflects the extracted features onto the corresponding mesh planes.

The remeshing module performs local mesh regeneration of the target region based on the features of the anomaly region extracted by the previous module. It comprises three components: arrangement, generation and grouping, and outputting. The arrangement component rearranges the feature points derived from the anomaly region and the original mesh model within the local mesh region based on their characteristics. The generation and grouping component generates new meshes based on the rearranged points, and groups the meshes accordingly. The outputting module outputs the updated meshes in the specified format for interaction with the FEA software.

### 2.2. Dataset

**Figure 2:**
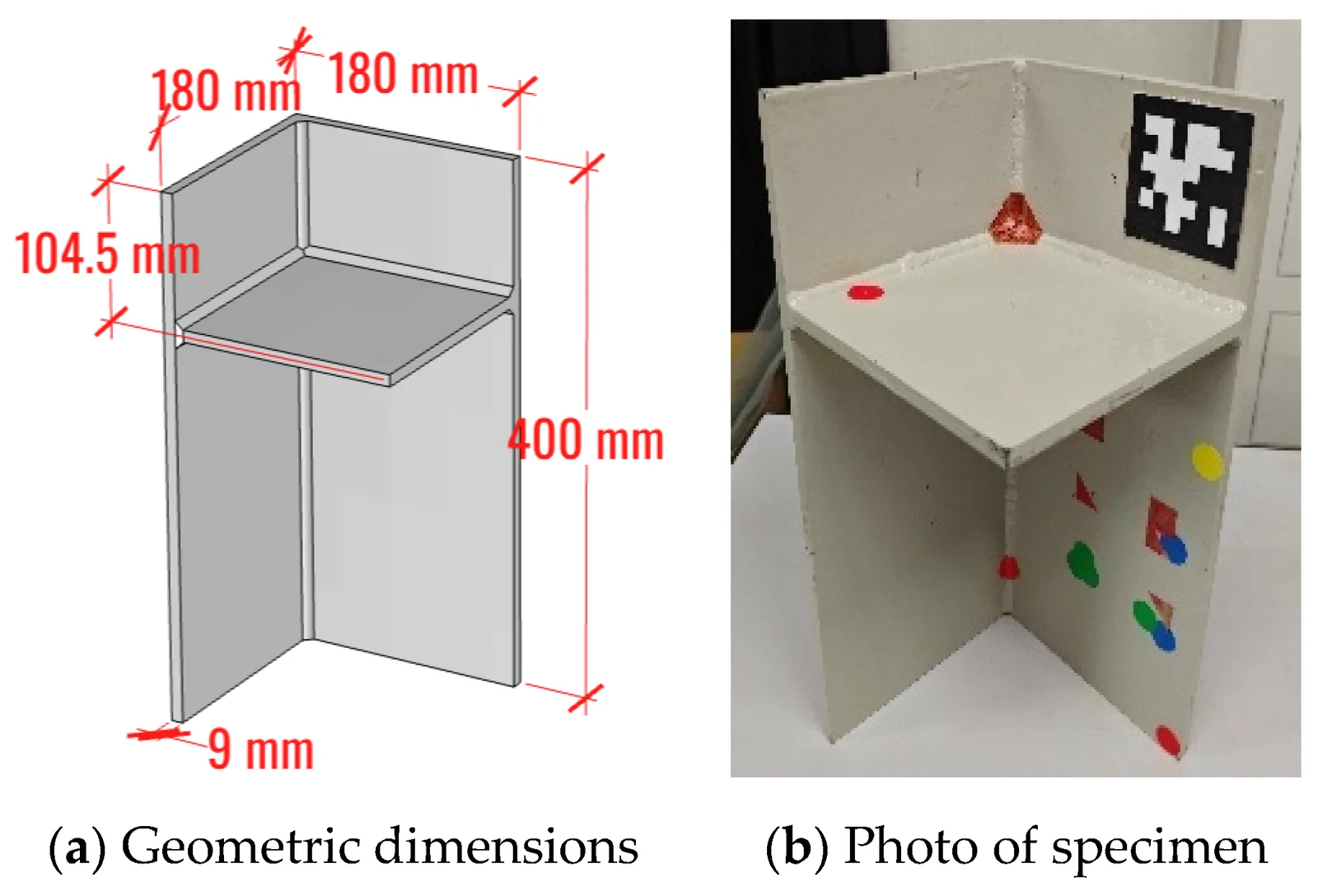
Experimental setup of the specimen.

The specimen used to evaluate the proposed framework is an angle-shaped steel member consisting of three steel plates joined at right angles. This specimen was selected because it represents a typical configuration found at joints and corners in steel structures, where damage, such as corrosion, commonly occurs. Figure 2 shows overviews of the angle-shaped steel specimen. It has outer dimensions of 400 mm in height, 180 mm in length, and 180 mm in width, and consists of three steel plates with a thickness of 9 mm welded at right angles. The surface was painted a light gray color.

Surface anomaly regions were created using colored stickers to simulate structural damage. Compared with actual damage, such as corrosion, the geometries of sticker-defined regions, including their shapes, boundaries, and areas, can be accurately determined from predefined parameters. This provides reliable ground-truth data for the quantitative evaluation of the updated model.

Actual damage often exhibits irregular geometries and may extend across multiple surfaces, particularly around joints and corners. Therefore, the anomaly regions were designed to represent a wide range of geometric characteristics that may be encountered in practice, including basic geometric shapes (e.g., circles, triangles, and rectangles), more complex shapes formed by combining these basic shapes, and regions spanning multiple surfaces.

**Figure 3:**
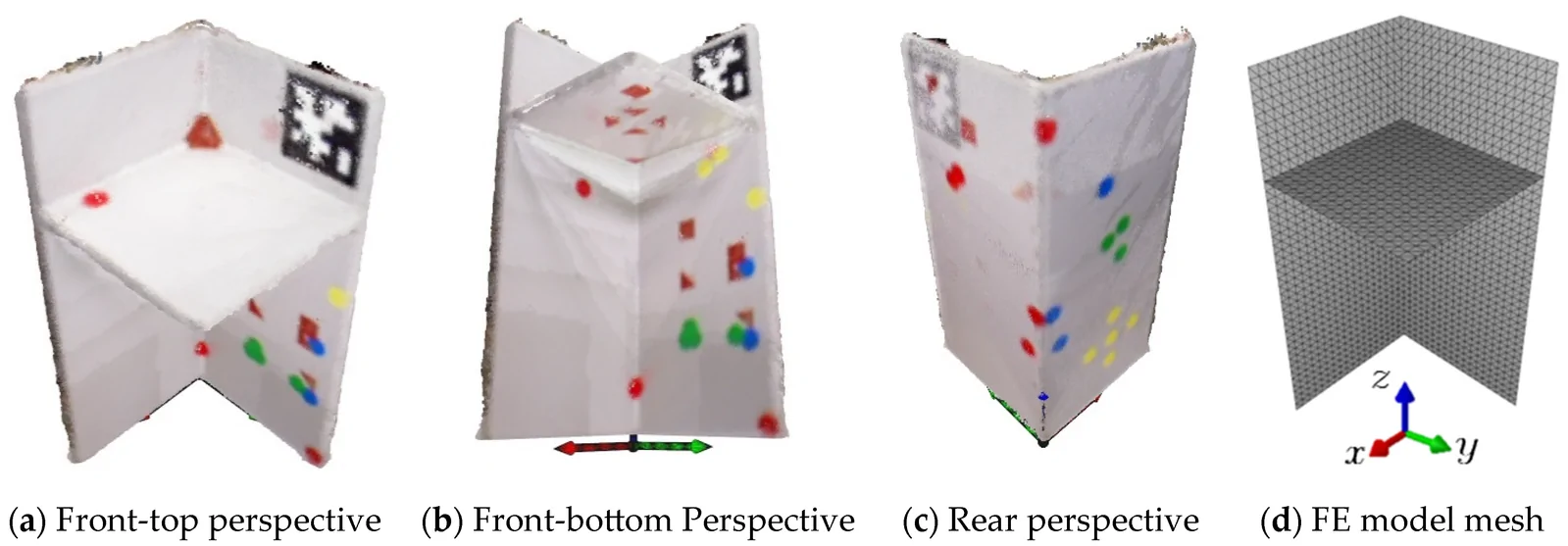
Acquired PCD data and nominal FE model mesh. In (**a**–**c**), the red, green, blue, and yellow represent sticker-defined anomaly regions used for color-based anomaly detection, while the black-and-white coded targets are AprilTags used by the scanning system as fiducial markers for loop closure and registration.

The PCD was captured in the laboratory using a handheld 3D scanner (DPI-8X, DotProduct LLC, Norwood, MA, USA). This device offers a measurement range of 0.3–1.9 m, with a standard accuracy of ±2 mm at a reference distance of 1 m. Additionally, it provides a resolution of 1.7 mm at 1 m. The specimen was placed vertically onto a small platform, with the end farther from the transverse stiffener (horizontal plate) facing downward. This positioning was intended to facilitate data acquisition near the lower region. The scanner was equipped with lighting to ensure complete capture of shaded areas during scanning. On the surface of the upper-right plane was a QR-code signal that served as a reference for the built-in registration of the scanner. The open-source software CloudCompare version 2.13.beta [25] was used to remove redundant parts and export the remaining parts in .pcd format, as shown in Figure 3a–c.

The shell-element FE models based on mid-plane meshes are commonly used for simulation of thin-walled structures whose thickness is much smaller than their in-plane dimensions. Therefore, a mesh model generated from the mid-plane geometry of the specimen plates was selected as the target model of the proposed updating framework, as shown in Figure 3d. This enables the updated geometry to be directly incorporated into the corresponding FE model.

### 2.3. Perception Module

#### 2.3.1. Registration Between the Surface Point Cloud and Shell-Element FE Model of a Same Structure

The PCD of the steel structure is registered to the mesh in two phases: rough and fine alignments. The principal component analysis (PCA) algorithm is used for coarse alignment, which ensures that the ICP algorithm [26] used for fine alignment can be performed smoothly.

The registration process used in this study was different from the typical point cloud registration, which aligns two point clouds of the same scale. However, to match the point cloud with the shell-element meshes, the shell-element model used here is generally based on the mid-plane position of the steel plate, which is generated from the point cloud of the surface of the structure. Therefore, each plane of the shell model must be aligned with the mid-plane of the corresponding point cloud. A point-to-plane ICP algorithm is used to align the point cloud and mesh by minimizing the distance from the point to the mesh face, as indicated by [27].

(1) $$E\left(R,t\right)=\sum_{i=1}^{N}{\left[{n_{i}}^{T}\left(Rp_{i}+t-q_{i}\right)\right]}^{2}$$

In Equation (1), **p***_i_* is the *i*-th point in the source point cloud, **q***_i_* is the corresponding point in the target point cloud, **n***_i_* is the normal vector at **q***_i_*, **R** is the rotation matrix, **t** is the translation vector, and *N* is the number of point correspondences. In this study, the plane points derived from the vertices of the meshes were used as the target point cloud, and **n***_i_* was the normal vector to the mesh plane to which the point belongs.

Additionally, PCA was implemented before ICP to align the two models approximately along their first three principal axes. However, for two sets of similar models, the corresponding axes could be reversed because of the random sign of the eigenvalues in the SVD computation. For a three-dimensional model, this leads to 2^3^ = 8 possible alignment results [28]. In this study, a method that solves the problem caused by the possible reversal of the principal axes was developed by comparing the overlap volume of the bounding boxes of the two models and selecting the result with the maximum overlap rate as the best.

(2) $$OR=\frac{V_{pcd}^{OBB}\cap V_{mesh}^{OBB}}{V_{mesh}^{OBB}}$$

**Figure 4:**
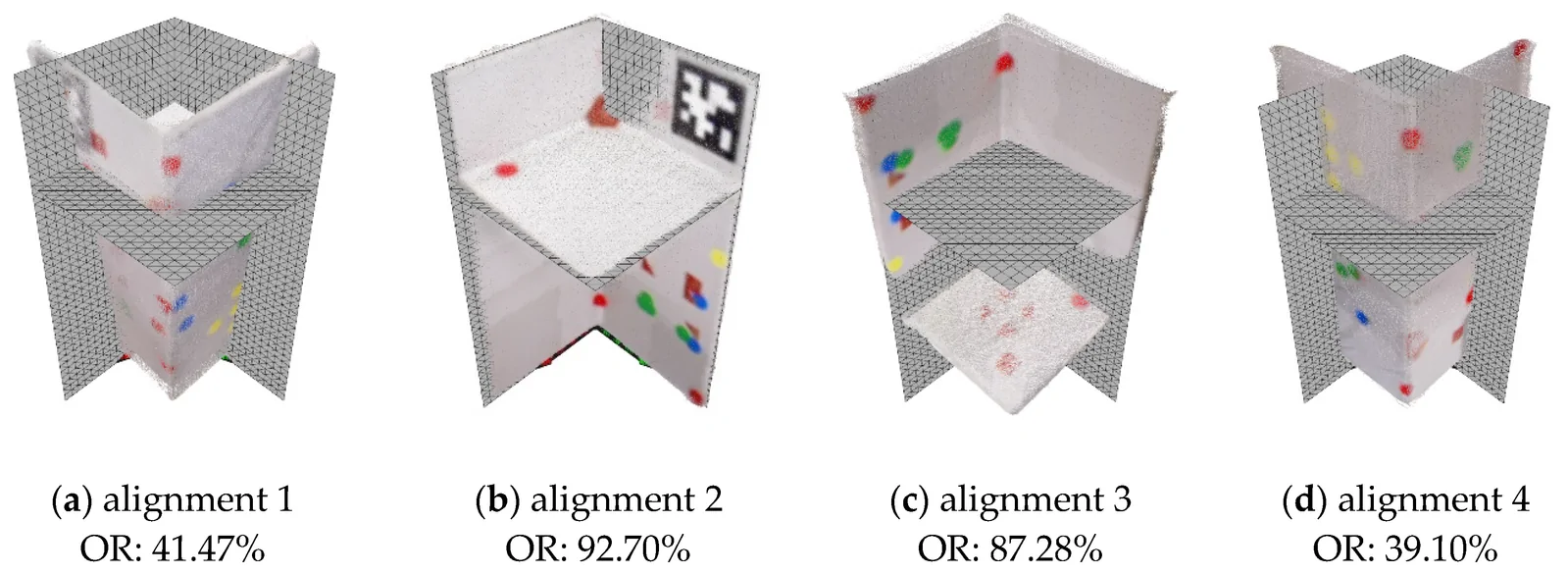
Optimization of four PCA alignments (OR: overlap rate).

In Equation (2), *OR* is the overlap rate of the two models, $V_{pcd}^{OBB}$ is the volume of the oriented bounding box of the point cloud, and $V_{mesh}^{OBB}$ is the volume of the oriented bounding box of the mesh. Flipping only the signs of the first two principal components and then calculating the third axis using the right-hand rule results in four valid right-handed coordinate system combinations during PCA alignment, as shown in Figure 4. Alignment 2, with an overlap rate of 92.70%, was selected as the optimal result.

**Figure 5:**
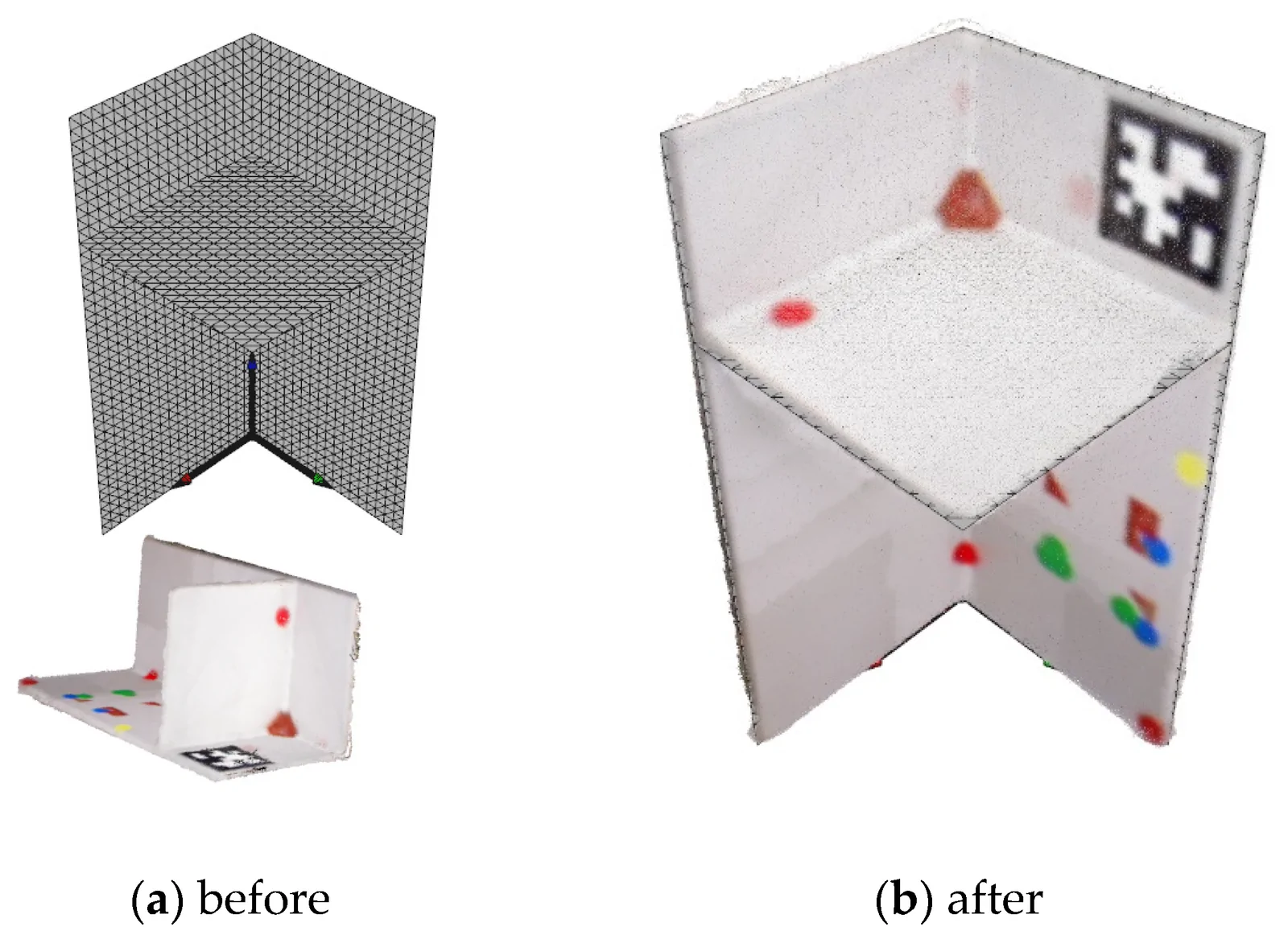
PCA + ICP registration.

The relative positions of the two models before and after registration are shown in Figure 5.

#### 2.3.2. Anomaly Detection Based on Color Information

A seven-dimensional feature vector **F***_color_* was constructed for foreground–background classification of regions exhibiting high color contrast. The foreground cluster was subsequently identified as the anomaly region associated with damage. Each component is defined as the product of a weight and color-space component. The weight represents the importance of the corresponding color-space component in the clustering process. Subsequently, K-means clustering is conducted on this feature vector space.

**Figure 6:**
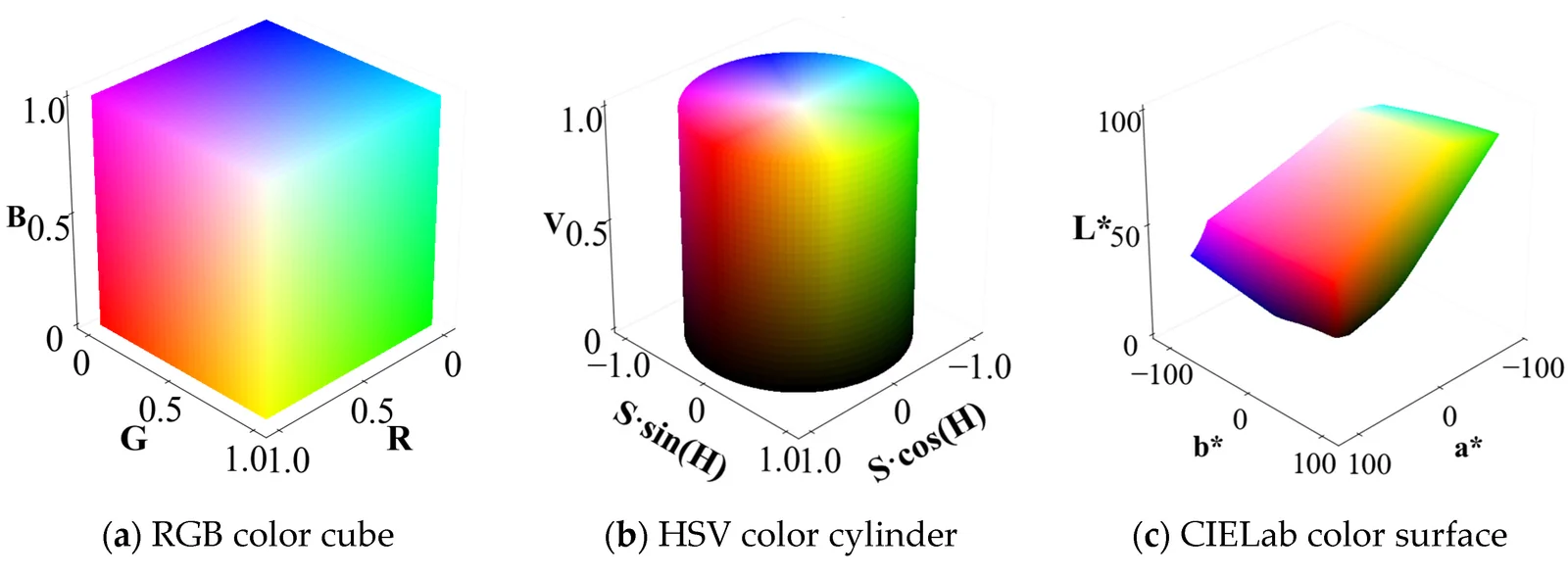
RGB, HSV, and CIELAB color spaces.

This feature vector incorporates seven values derived from three different color spaces (RGB, HSV, and CIELab, as shown in Figure 6) to fully leverage their respective advantages in representing the color characteristics. The RGB values are directly extracted from the color information of the point cloud, expressing color as *R* for red, *G* for green, and *B* for blue. The HSV and Lab values are derived through color-space conversion from RGB. The HSV model expresses color as *H* for hue, *S* for saturation, and *V* for value. The Lab model expresses color as L* for perceptual lightness and a* and b* for the four unique colors of human vision: red, green, blue, and yellow. The weights control the relative influence of the color components on clustering. The R and G components represent the red and green channel intensities, respectively; increasing their weight emphasizes differences in these intensities but may also increase the influence of brightness variations. The sine and cosine of H provide a continuous circular representation of hue, and increasing their common weight emphasizes hue differences. The S component represents color saturation, so a larger weight increases sensitivity to saturation differences. Similarly, increasing the weights of b* and a* emphasizes blue–yellow and red–green differences, respectively. The V and L* components represent value and perceptual lightness, respectively, and were excluded to limit the direct influence of brightness differences on clustering.

Based on these considerations, an initial weight combination was selected empirically and then iteratively adjusted through visual inspection of the extraction results. This process aimed to obtain a common set of weights for extracting the different sticker colors and their combinations in the presence of local brightness variations in the acquired point cloud. Among the combinations examined, the selected weights yielded the most complete foreground extraction, as judged by visual inspection, and were adopted for subsequent model updating. The feature vector **F***_color_* was finally defined as shown in Equation (3).

(3) $$F_{color}={\left[0.3G,0.3R,1.5\cos\left(H\right),1.5\sin\left(H\right),2.5S,2.0b^{*},1.0a^{*}\right]}^{T}$$

**Figure 7:**
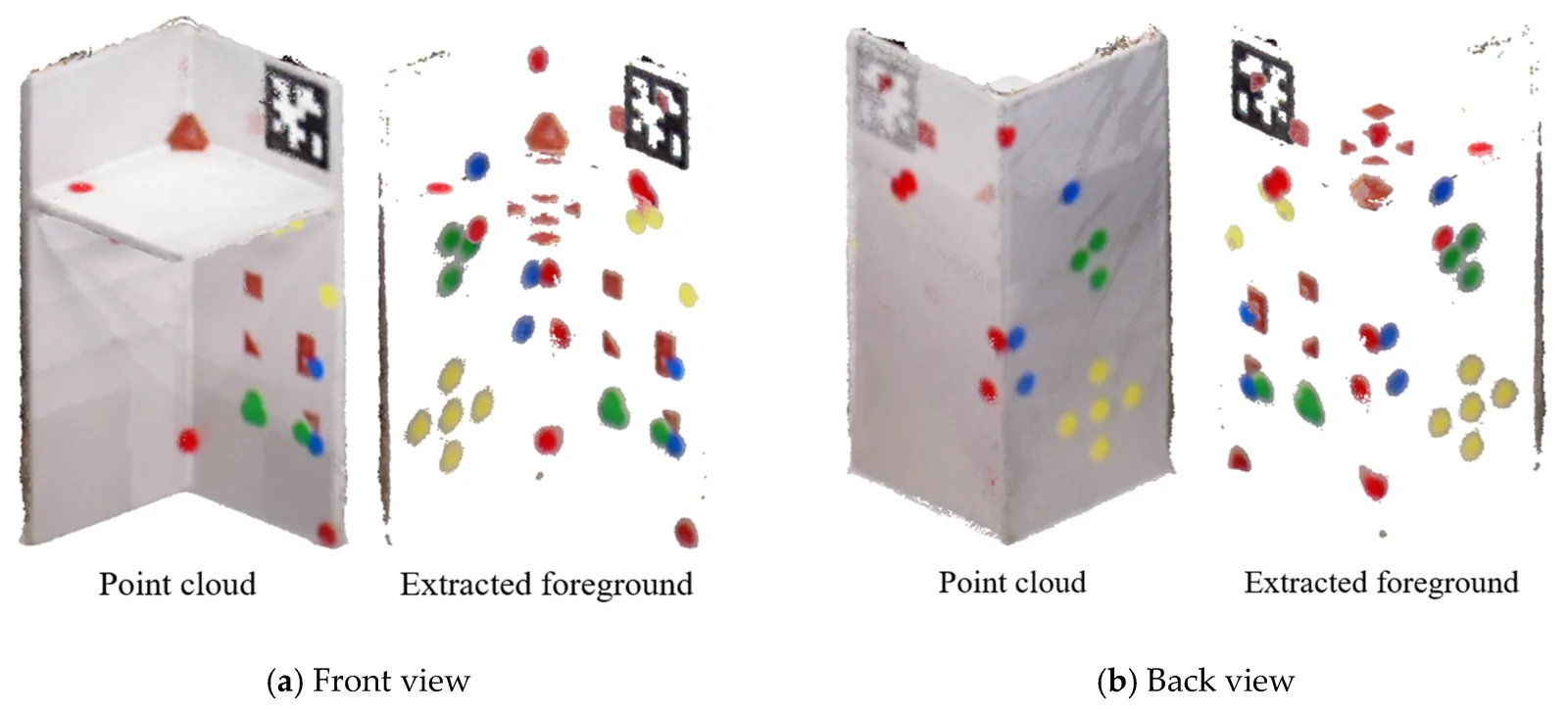
Foreground extraction using seven-feature K-means clustering: original point clouds and extracted foreground points shown in front and back views. The red, green, blue, and yellow represent sticker-defined anomaly regions used for color-based anomaly detection

The anomalous point extraction is shown in Figure 7. Some of the extracted dark regions could also be manually removed. In this step, the detected points qualitatively match well with the anomaly regions. The method consistently identified red, yellow, blue, green, and black foreground regions with high contrast against the gray-coated surface. Visual inspection indicated that foreground identification was maintained despite local brightness variations in the acquired point cloud. However, the stability of the selected weights under independently varied lighting conditions needs systematic evaluation.

### 2.4. Anomaly Description Module

#### 2.4.1. Edge Extraction of Anomaly Regions

In the proposed method, boundary points are extracted to represent individual anomaly regions and are referred to as feature points. Therefore, detecting these boundary points is required. Considering that the point cloud is distributed on the surface of the structure and that infrastructure structures generally consist of flat surfaces, a two-dimensional Alpha-shape algorithm [29] was selected to detect the boundaries of the coplanar point set in the individual anomaly regions. The Alpha-shape algorithm assumes an open disk with a radius equal to a positive real number α. Let S be a finite set of points in the plane. An open disk is considered empty if it contains no points of S. The α-hull of S is defined as the set of points that are not contained in any empty open disk of radius α. The boundary of the α-hull comprises circular arcs of constant curvature 1/α, whereas the α-shape denotes the α-hull that uses straight lines instead of circular arcs.

Based on this concept, the first step in implementing the algorithm typically involves computing the Delaunay triangulation of the point set. A triangle is retained in the *α*-shape if and only if the radius of its circumcircle is less than or equal to a predefined threshold determined by parameter. The definition states that the smaller the alpha, the more details that can be captured by boundary detection. However, this can lead to overfitting. In this study, to obtain the most accurate boundaries possible, a packaged optimization algorithm was applied to select an optimal alpha value for each point set through 100 iterations of computation and evaluation, with the alpha value ranging from 0.01 to 3. The algorithm tends to select the smallest value that covers all points to maintain the details of the boundary. To accelerate this process, the attempt is executed three times for every point set, each time on a randomly downsampled (40% left) subset of the projected 2D points.

The detected boundaries are initially simplified using the Ramer–Douglas–Peucker algorithm [30,31]. This algorithm, which is given a sequence of ordered points and a user-defined tolerance *ε*, begins by connecting the first and last points of the curve with a straight line. Subsequently, it identifies the point that deviates the most from this line, as measured by the perpendicular distance. If the maximum deviation exceeds *ε*, this point is considered essential to the shape, and the curve is recursively split at this location. The algorithm is applied to each resulting segment until all points lie within *ε* from their corresponding segment lines.

Finally, B-spline curve processing is used for curve smoothing to reduce overfitting. The B-spline curve is defined as Equation (4):

(4) $$C\left(u\right)=\sum_{i=0}^{n}N_{i,p}\left(u\right)\cdot P_{i}$$

where *C*(*u*) denotes a point on the B-spline curve at parameter value *u*, *P_i_* denotes the control points, $N_{i,p}\left(u\right)$ are the B-spline basis functions of degree *p*, and *n* + 1 is the number of control points.

In the base case, corresponding to degree 0, the B-spline basis function is defined as in Equation (5):

(5) $$N_{i,0}\left(u\right)=\left\{\begin{array}{cc} 1 & u_{i}\leq u\leq u_{i+1}\\ 0 & \mathrm{otherwise} \end{array}\right.$$

Higher-order B-spline basis functions are recursively defined as in Equation (6):

(6) $$N_{i,p}\left(u\right)=\frac{u-u_{i}}{u_{i+p}-u_{i}}N_{i,p-1}\left(u\right)+\frac{u_{i+p+1}-u}{u_{i+p+1}-u_{i+1}}N_{i+1,p-1}\left(u\right)$$

where *u_i_* denotes the knot values from the nondecreasing knot vector $U=\left\{u_{0},u_{1},\ldots ,u_{m}\right\}$, and the terms with zero denominators are defined as zero. *m* + 1 is the total number of knots, which satisfies Equation (7):

(7) m=n+p+1

**Figure 8:**
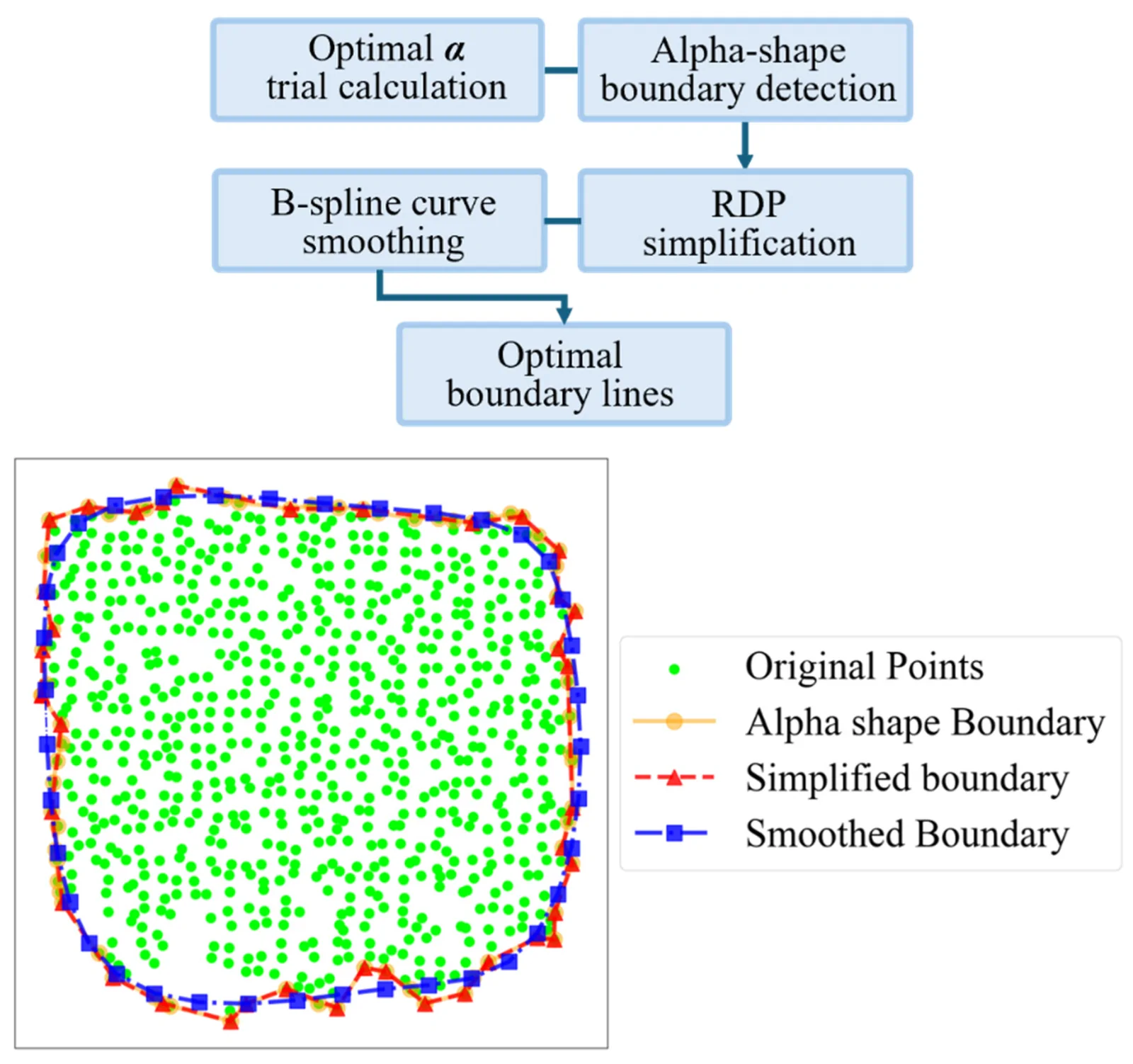
Optimal Alpha-shape boundary detection.

All steps are illustrated in Figure 8 with an example. Here, green dots are the extracted anomalous points, and the orange line, marked with single dots, indicates the raw *α*-shape with the optimal alpha, which may reflect excessive details. The red dotted line, marked with triangles, indicates the simplified boundary, which is recursively simplified with *ε* of 0.2 mm. The dot-dashed line, marked with square markers, indicates the final boundary after being smoothed by Equation (4) with a cubic B-spline (*p* = 3). This constitutes an intermediate step in remeshing, the performance of which is evaluated against the ground truth of the anomaly regions after remeshing, as described in Section 3.3.

#### 2.4.2. Categorization by Planar Clustering

The method of representing anomaly regions by their detected boundaries, as well as the subsequent remeshing approach, is based on the assumption that all target points are coplanar. However, a single damage region that extends across multiple surfaces is commonly observed in practice. Therefore, the extracted anomalous point set extending across multiple surfaces is partitioned into coplanar point subsets according to their corresponding planes, allowing the processing for coplanar point sets to be applied to each subset. Meanwhile, feature points along the shared boundaries between adjacent point subsets are matched to ensure mesh continuity after remeshing.

**Figure 9:**
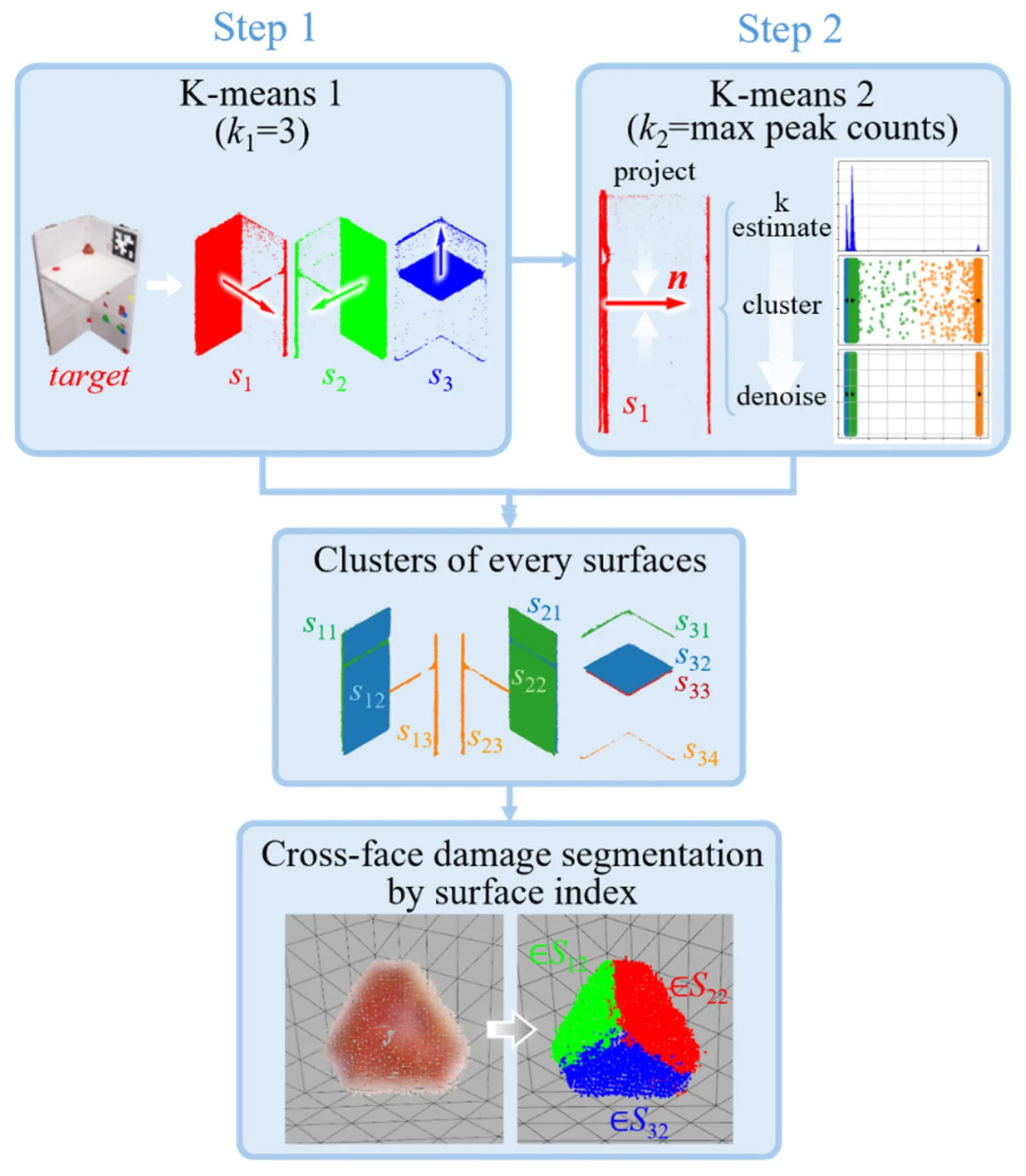
Point separation based on planar clustering. The five-pointed star markers shown in Step 2 denote the cluster centroids.

The entire flow of clustering is shown in Figure 9. Here, the first-step clustering is performed using K-means on the normal vectors of points. All points are clustered based on the orientation of the planes to which they belong. The cluster number $k_{1}$ is assigned as 3 for three-dimensional point cloud. The resulting clusters are denoted as $S_{k_{1}}$, where $k_{1}$ denotes the cluster label obtained from the first-step clustering. For each cluster $S_{k_{1}}$, the points are distributed across a set of parallel planar surfaces sharing the same normal direction $n_{k_{1}}$. In the second-step clustering, all points in cluster $S_{k_{1}}$ are projected onto the corresponding normal axis $n_{k_{1}}$, and secondary K-means clustering is performed on the resulting one-dimensional projection values to separate the points into different planar clusters. Here, the number of clusters is set to $k_{2}$, which is determined by the number of local maxima in the histogram of point projections along the normal direction $n_{k_{1}}$. The resulting clusters for $S_{k_{1}}$ after denoising are denoted as $S_{k_{1}k_{2}}$, where $k_{2}$ denotes the cluster label obtained from the second-step clustering and denoising. Denoising was performed using iterative 3σ clipping along the normal direction $n_{k_{1}}$, with the iteration terminated when the cluster centroid remained unchanged between successive iterations Each cluster contains all the points on a single planar surface of the structure. Based on the clustering results, the points of a single anomaly region can be separated using clustering labels.

### 2.5. Remeshing Module

#### 2.5.1. Feature Point Arrangement for Coplanar Point Sets

**Figure 10:**
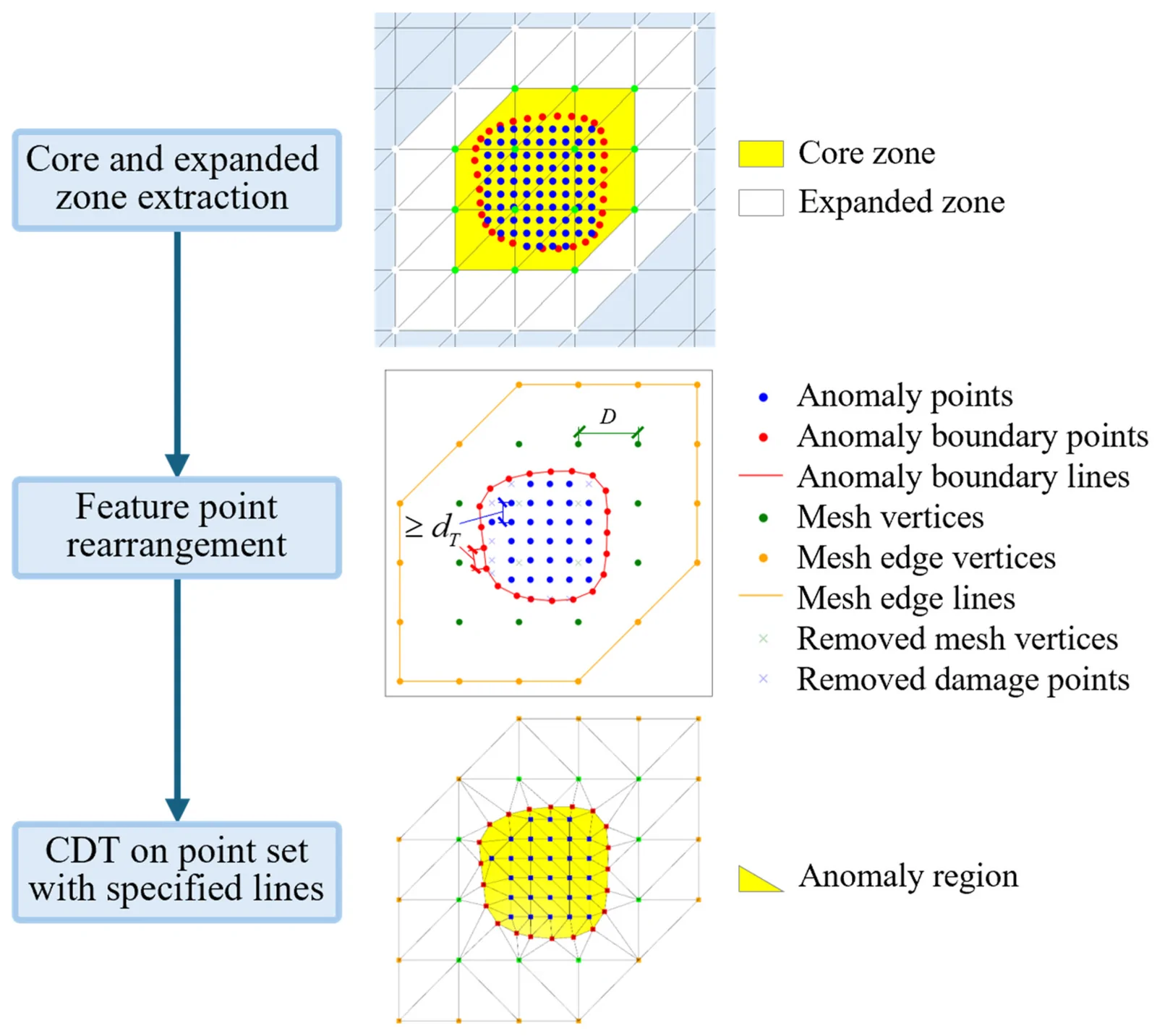
Rearrangement of feature points.

In this study, the process of model updating with damage information essentially involved remeshing a corresponding region in the original mesh according to the feature of the extracted anomaly region. The primary challenge in this process was to obtain a proper remeshing result that fully considers the shape and relative size of the anomaly region against the original mesh. For this purpose, the Constrained Delaunay triangulation (CDT) algorithm [32] was applied to the remeshing region, which was treated as a domain combining a point set and non-crossing line segments in the plane, known as the planar straight-line graph. The remeshing flow is illustrated in Figure 10. CDT is a classical triangulation of vertices that includes the pre-specified edges and approximates the Delaunay triangulation as closely as possible, which is advantageous for FEA. In this study, the remeshing region was extracted based on the boundary detection of the anomaly region, and the points inside this remeshing region were rearranged by referring to the vertices of the original mesh. To maintain the outline of the anomaly and remeshing regions, constrained edges were assigned to the remeshing region and the boundaries of the anomaly region and were subsequently included in the triangulation by the CDT.

In the theory of the FEA method, it is well known that the shape of triangular elements significantly affects convergence and accuracy, and the equilateral triangle is the ideal shape of elements. However, in engineering applications, strictly conforming to such ideal elements often leads to over-meshing near regions with geometric discontinuities caused by damage. This can adversely affect convergence performance and computational efficiency because of the increased number of elements.

To address this issue, a non-Steiner triangulation is assigned, in which only the input points can be used, and no extra vertices are allowed. Given that the FE model of an intact structure can be evenly re-meshed, the average distance *D* from each original vertex within the remeshing region to its two nearest neighbors was computed. A threshold distance *d_T_*, defined as one-third of the average of all *D* values across the entire remeshing region, was used to control the size ratio between adjacent elements around the anomaly region to below three. The feature point rearrangement procedure is detailed as follows: The feature points (i.e., the boundary points of the damage region) are extracted. All original meshes containing vertices that fall within the closed polygon formed by these boundary points are extracted as the core target for remeshing. An additional mesh layer is expanded outwards from the core target to provide a buffer zone for the transition of the mesh size. The core target and expanded layer constitute the remeshing region. Within this region, the original vertices of the mesh, along with the boundary and sampling points inside the closed polygon formed by the boundary points, are considered remeshing vertices. The original vertices that fall within the closed polygon formed by these boundary points or are excessively close (less than *d_T_*) to the boundary are removed. The boundary points are also down-sampled to the space of *d_T_* while preserving the boundary shape. Inside the boundary, points are uniformly sampled within the space of *d_T_*, and sampled points that are excessively close (less than *d_T_*) to the boundary are removed.

#### 2.5.2. Feature Point Alignment Along Joint Line for Damage Extending Across Multiple Surfaces

For damage extending across multiple surfaces, after clustering using the method described in Section 2.4.2 (Categorization by Planar Clustering), each subset of a single anomaly region can be treated as a coplanar point set. The same approach can be applied for boundary detection and extraction, as well as for projection onto the original mesh plane, enabling the extraction of the remeshing zone and rearrangement of the remeshing vertices.

However, to ensure that the regenerated meshes on different planes share common nodes at their joints, the corresponding feature points of every subset on different planes must be matched along the joint lines during the point rearrangement.

**Figure 11:**
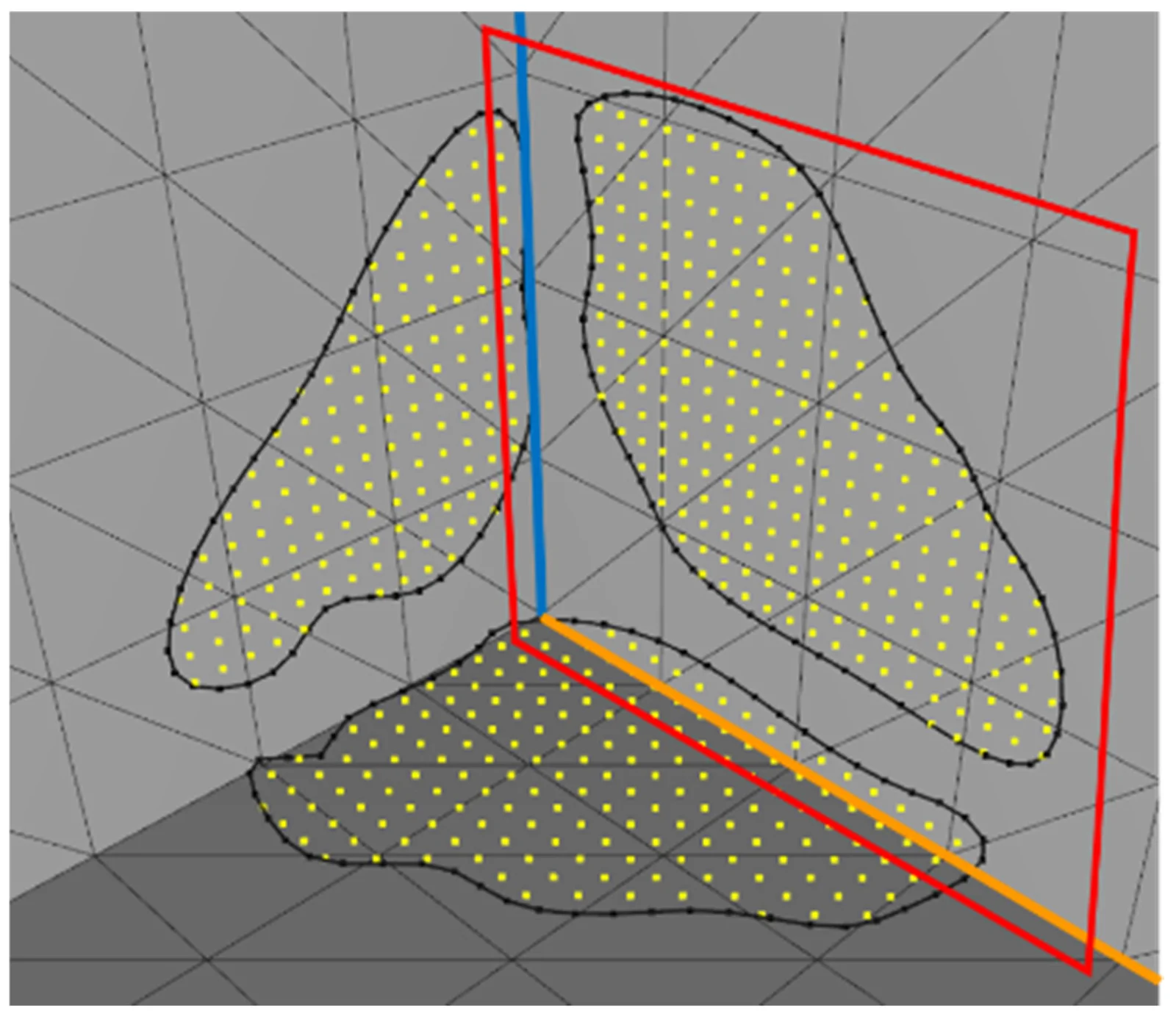
Target subset of rearrangement.

**Figure 12:**
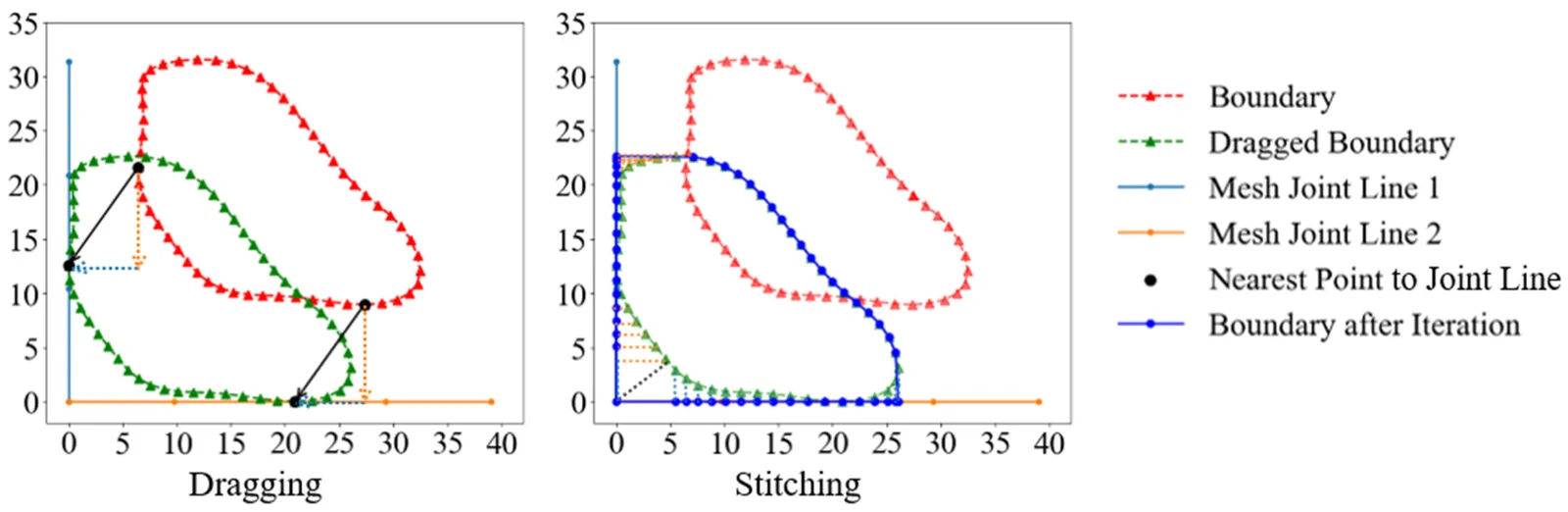
Dragging and stitching of anomaly-boundary points onto the mesh joint lines during cross-surface boundary alignment.

**Figure 13:**
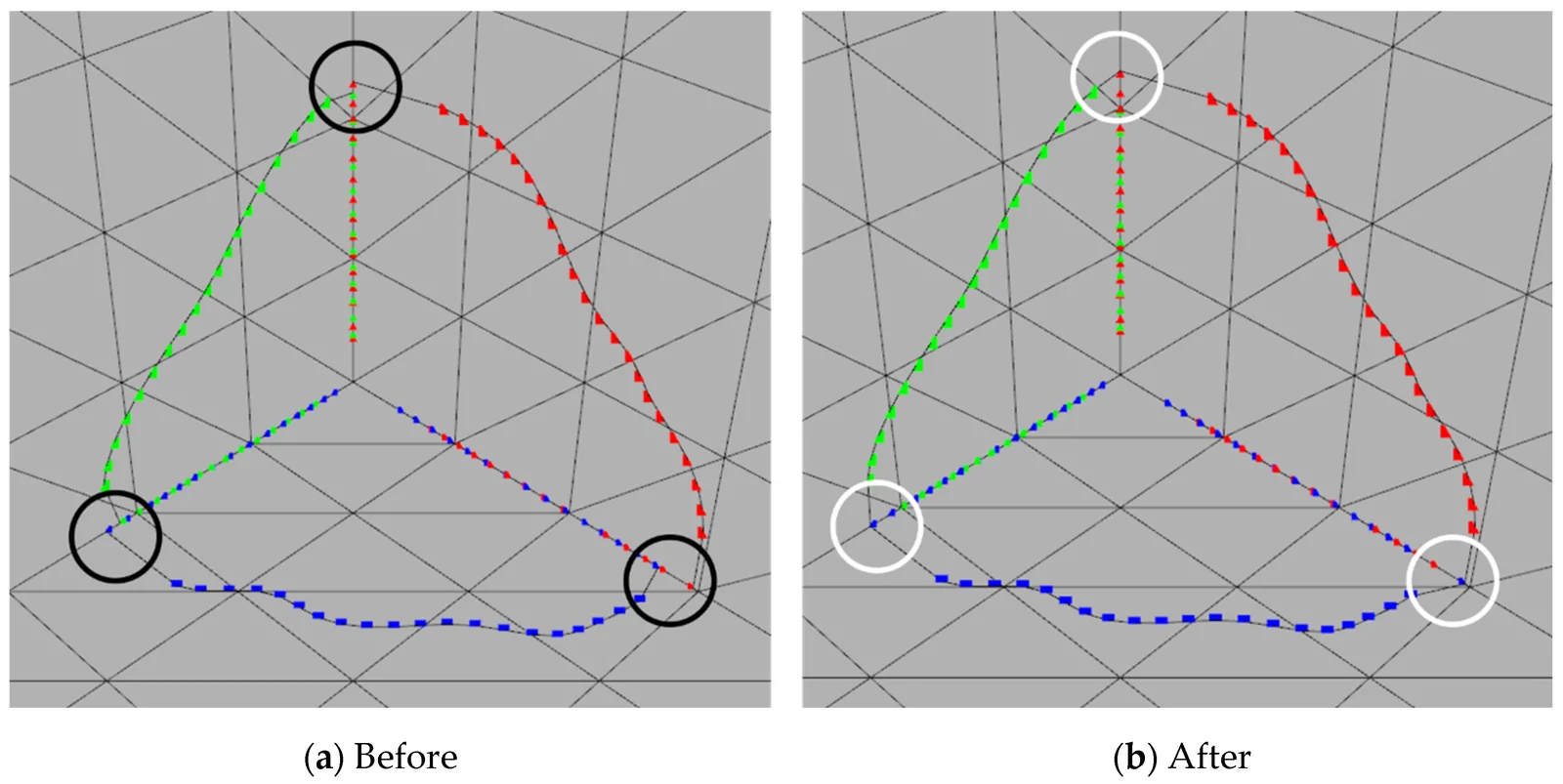
Match joint lines.

Consider the damage extending across three surfaces as an example. The same approach is also applicable for the damage extending across two surfaces. After separating the anomaly-region points into subsets corresponding to each plane, the boundary points in each subset are extracted and projected onto the respective mesh plane, as shown in Figure 11. An alternating iterative process is then applied to shift the boundary point nearest to the joint line onto the line until the area enclosed by the boundary no longer increases. This process registers the feature points to the correct positions on the mesh plane, which is referred to as “Dragging and stitching”, as shown in Figure 12. Accordingly, a stitched boundary is obtained. The stitched boundary points on the joint line are matched between different subsets to ensure that the newly generated meshes from the respective planes are consistent and share the same vertices along the joint line, as shown in Figure 13.

### 2.6. Summary of Processing Parameters

The principal processing parameters are summarized in Table 1. The same boundary-extraction settings were used for the 13 anomaly point sets and sub point sets obtained from the ten anomaly regions, while the final α value was determined separately for each point set.

No fixed random seed was specified for down-sampling. Qualitatively consistent reconstruction results were observed across repeated runs with the same processing settings. However, run-to-run variability was not quantitatively evaluated, and sensitivity to random sampling and processing parameters remains to be systematically assessed.

## 3. Performance Evaluation of Updated Model

The proposed framework was evaluated using a simulated specimen with predefined anomaly regions representing damage. After introducing the anomaly region configurations and the FEA setup, the updated model generated by the framework was evaluated from two perspectives. The geometric accuracy of the updated model was quantified by comparing the reconstructed anomaly regions with their designed geometries using the Intersection over Union (IoU). Furthermore, the performance of the updated model in FEA was evaluated by comparing its structural responses with those of a manually constructed reference model under identical conditions.

### 3.1. Case Settings of Anomaly Regions

**Figure 14:**
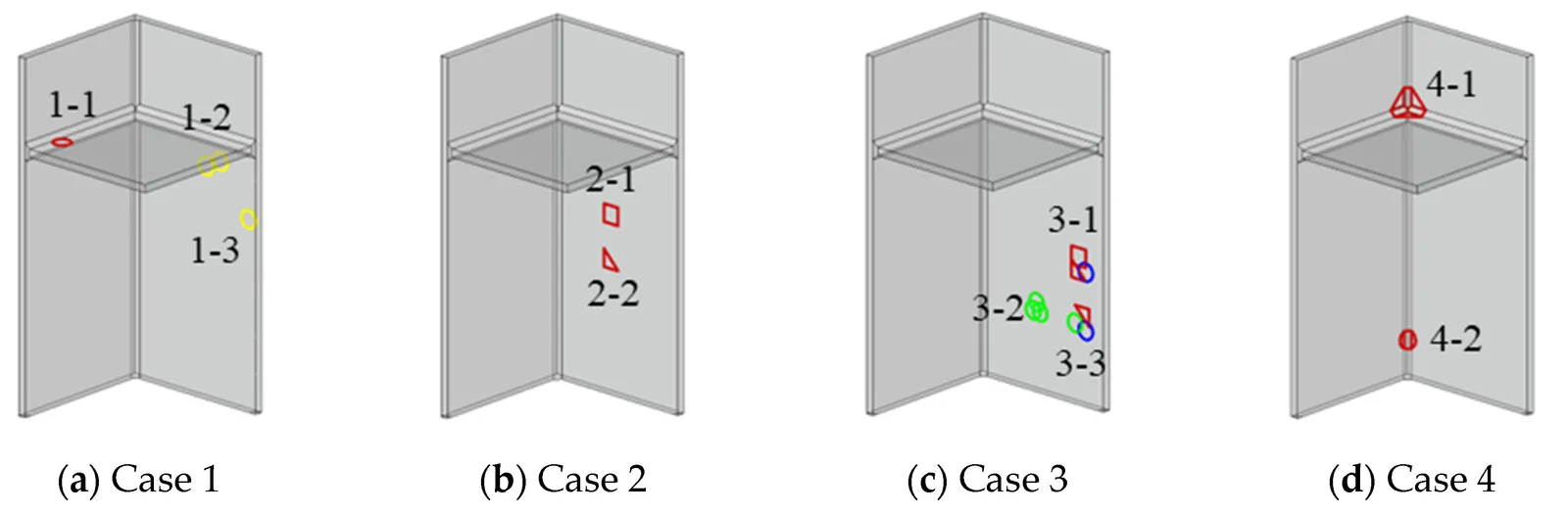
Case arrangements.

In this study, artificially introduced anomaly regions were employed to simulate anomalous surface conditions caused by structural surface damages. The diverse and randomly shaped surface damage regions encountered in practice were idealized into several representative geometric features. Accordingly, the artificial anomaly regions were categorized into four major groups based on these features. To assess the generalizability of the proposed framework to diverse damage geometries, the anomaly regions were designed to cover these representative geometric features through three fundamental shapes—circular, triangular, and rectangular—as well as their combinations. The case settings of anomaly regions are shown in Figure 14. Four categories comprising ten cases were designed to cover the representative geometric features discussed above.

Case 1 represents damage regions with smooth convex boundaries at different locations. Case 1-1 is positioned near the intersection of steel plates. Owing to the geometric simplification introduced by the shell-element representation at plate intersections, the projected location of the damage region on the mid-plane may inevitably deviate from its actual position, and the influence of this deviation on the analysis results is therefore investigated. Case 1-2 consists of two closely spaced anomaly regions. In practice, color blurring and ghosting effects in point-cloud data may make it difficult to distinguish nearby damaged regions; this case was designed to assess the impact of such situations. Case 1-3 places the anomaly region near a plate edge, where point-cloud quality is generally lower and damage localization is more susceptible to positional errors.

Case 2 represents damage regions with boundaries containing sharp corners. Case 2-1 contains four right-angle corners, whereas Case 2-2 further includes two acute-angle corners. These cases were designed to evaluate the capability of the proposed method to capture sharp geometric features along damage boundaries.

Case 3 employs combinations of basic geometric shapes to represent larger damage regions and more complex geometric features. Case 3-1 contains an internal hole, Case 3-2 includes obtuse-angle concave features along the boundary, and Case 3-3 contains a high-aspect-ratio boundary concavity.

Case 4 represents damage regions extending across multiple surfaces. Case 4-1 simulates a damage region extending across three surfaces around a corner, whereas Case 4-2 simulates a damage region spanning across two surfaces at the intersection line between steel plates.

The specimen surface was coated with gray paint, representing the light-colored coatings commonly adopted in practice to facilitate the visual identification of surface damage through color contrast. Since the proposed anomaly-detection procedure is based on foreground–background classification rather than the identification of a specific damage color, the artificially introduced anomaly regions were assigned four highly saturated colors: red, green, blue, and yellow. These colors were selected to represent a range of extreme color contrasts with respect to the background coating and to evaluate the robustness of foreground–background classification under different high-contrast conditions. It should be noted that the objective of this study is not to model the color characteristics of specific damage types, but to evaluate the capability of the proposed framework to extract and represent anomaly-region geometries under various high-contrast foreground–background conditions.

### 3.2. FEA Setup

**Figure 15:**
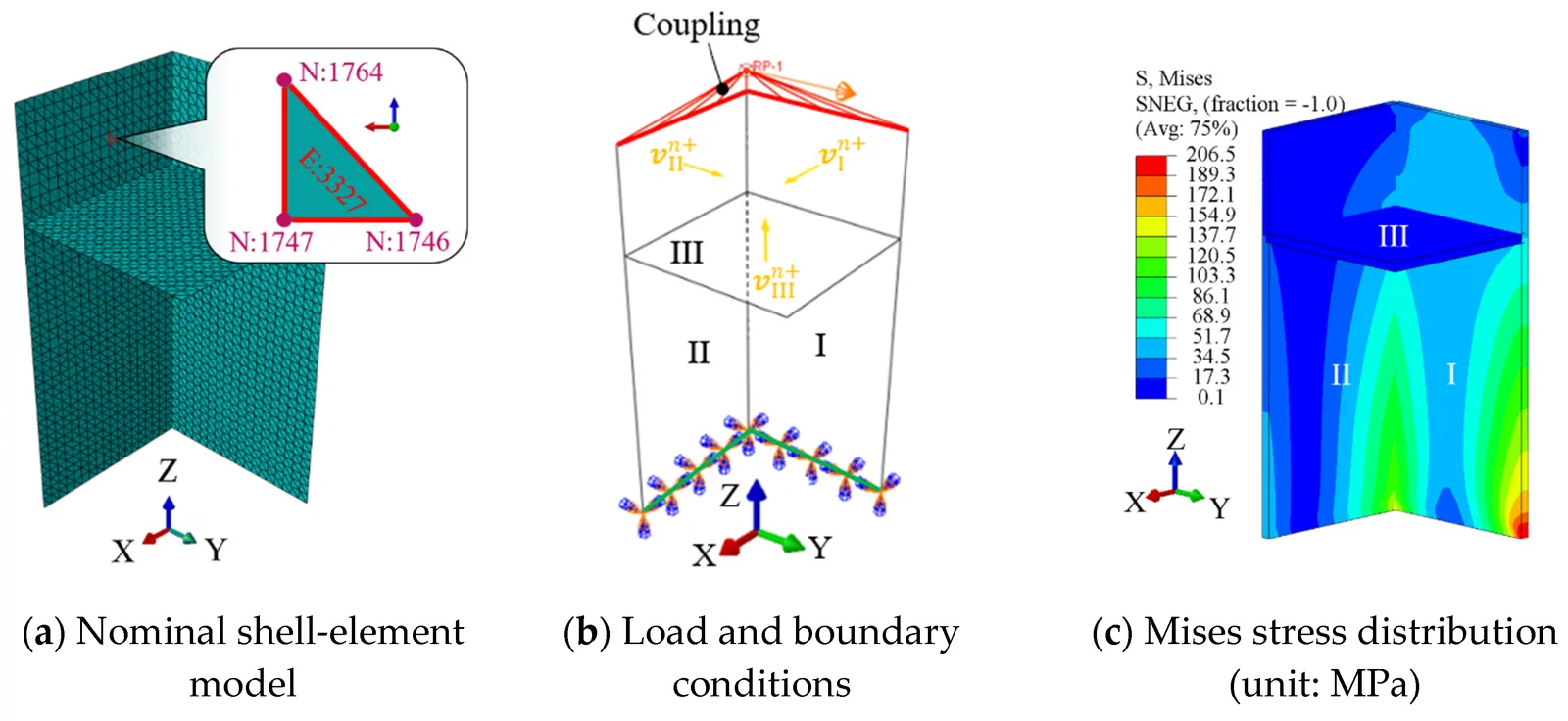
Original FE model and elastoplastic analysis under static loading.

Finite element analysis was conducted using the general-purpose software Abaqus/CAE version 2022. As shown in Figure 15a, the linear geometric order three-node triangular shell elements (S3 type) with the global control of mesh size of 10 mm were generated. The total number of elements was 3528 for the original model. The mechanical properties of the steel material SS400 were assigned for elastic–plastic static analysis, with a Young’s modulus of 206 GPa, Poisson’s ratio of 0.3, and yielding stress of 300 MPa. The stress–strain curve of the plastic region to be assigned was created by fitting the given curve to the SS400 material data sheet. The boundary condition was set up at the bottom edge lines with all DOF fixed. A static distributed load was applied at the top edge lines by providing a *y*-directional displacement of 8 mm at a reference point offset from the top joint point of the specimen by 20 mm and connected with the top edge lines by a coupling constraint, as shown in Figure 15b. Specifically, the specimen was subjected to global uniaxial cantilever bending, which represents a fundamental loading case in thin-walled members in which tension and compression occur along the same axis.

Figure 15c shows the stress distribution during the elastic phase under a total loading of 0.7 mm. The stress gradually increases from top to bottom, which is consistent with the loading pattern of a cantilever beam fixed at the bottom. In Plate-I, in-plane bending dominates the structural response. Owing to the constraint imposed by the transverse stiffener, out-of-plane stress develops only near the region located approximately one-third of the plate height from the bottom. In contrast, out-of-plane bending dominates the structural response of Plate-II. The high-stress region concentrates at the lower-right corner of plate I and at the lower extremity of the joint line connecting the two plates.

### 3.3. Geometric Accuracy of the Updated Model

**Figure 16:**
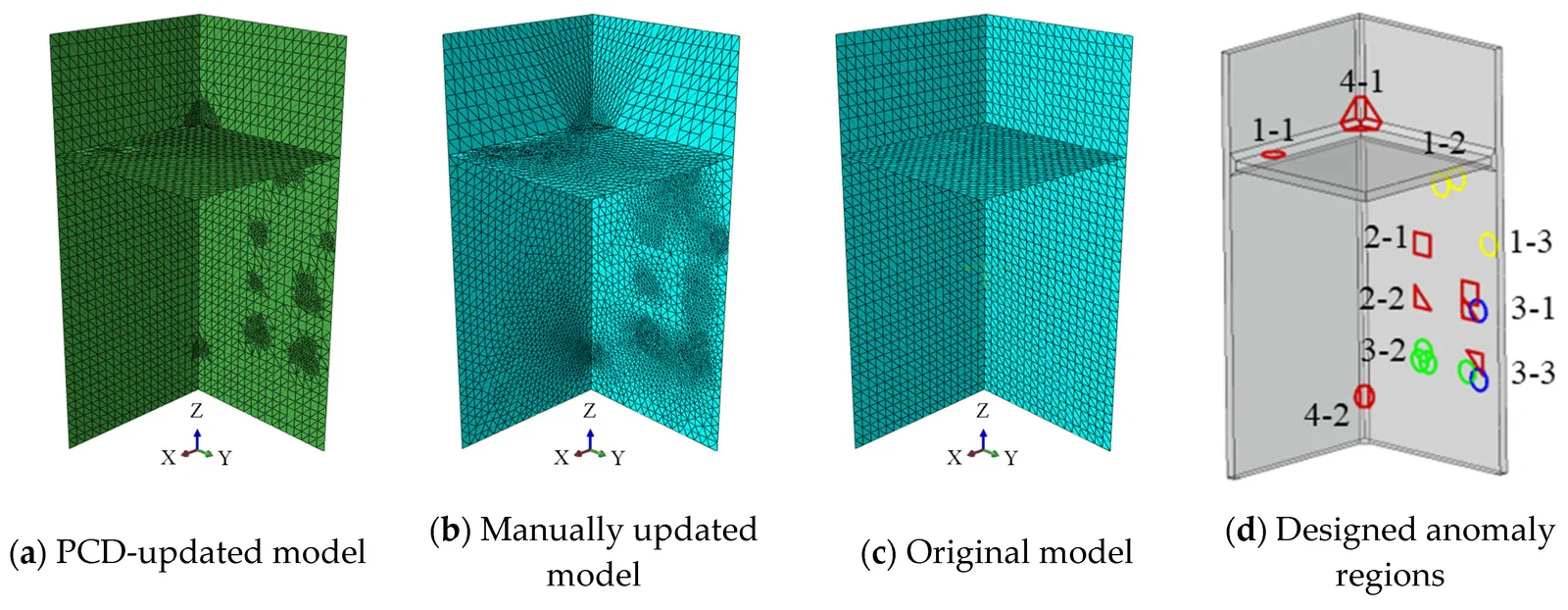
Updated FE model and comparisons.

Figure 16 presents the overall results of the updated and original models, alongside the design drawing. The manually updated model was re-meshed on the updated geometric model with imprinted designed boundary lines of anomaly regions. The local seeds were assigned along the boundary lines at intervals of one-third of the original mesh size. And free triangular meshing was used for the manually updated model so that all three models used S3 shell elements. Differences in mesh density and topology nevertheless remained. The mesh characteristics of the three models are shown in Table 2.

**Figure 17:**
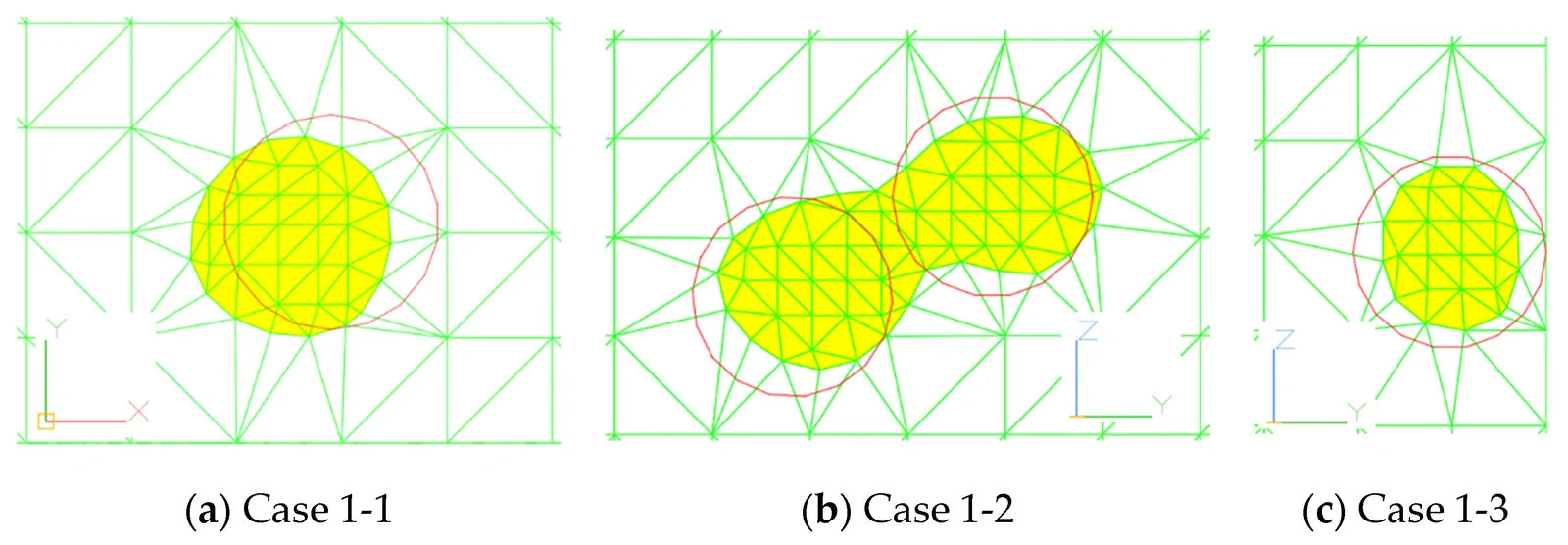
Updated mesh results of Case 1.

**Figure 18:**
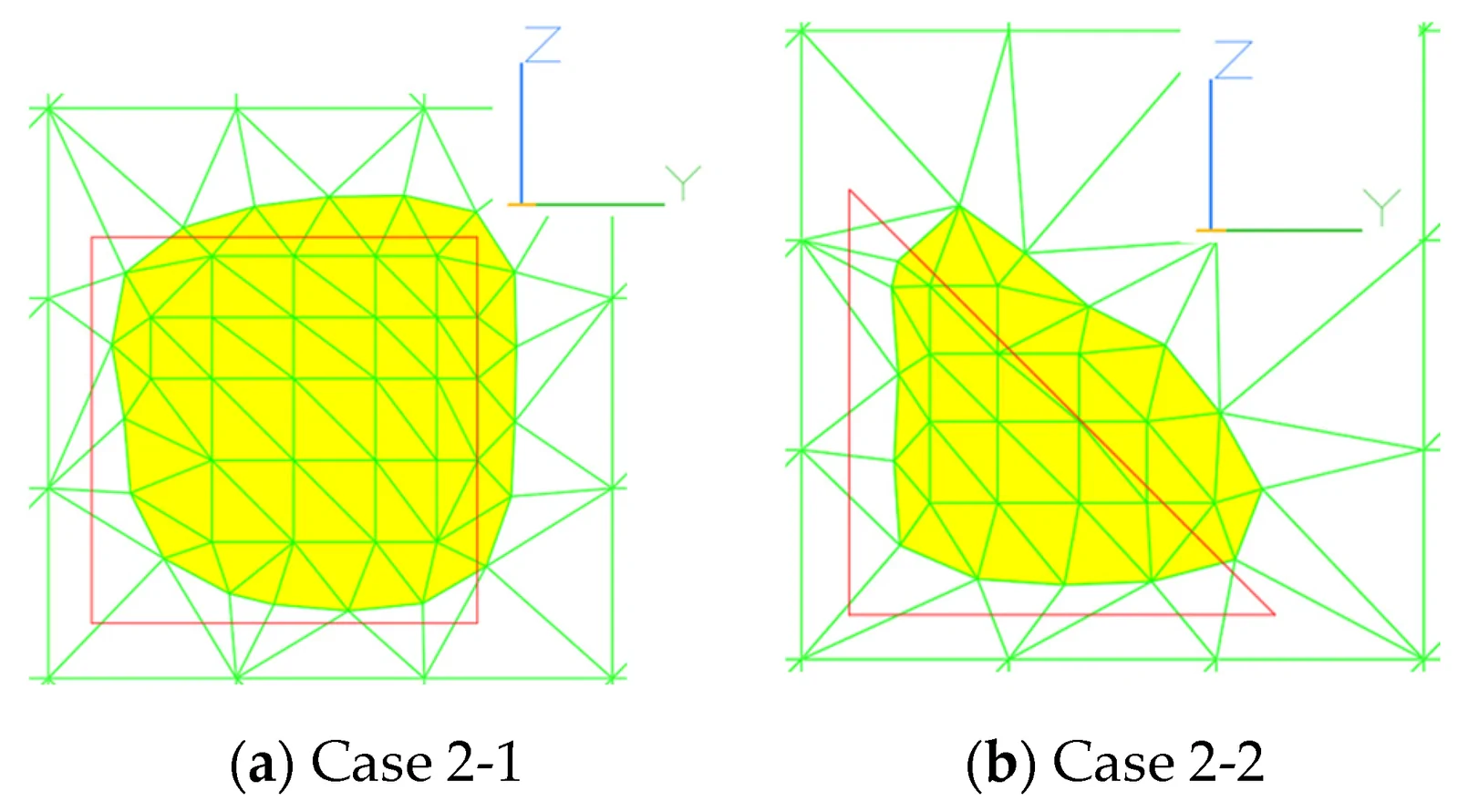
Updated mesh results of Case 2.

**Figure 19:**
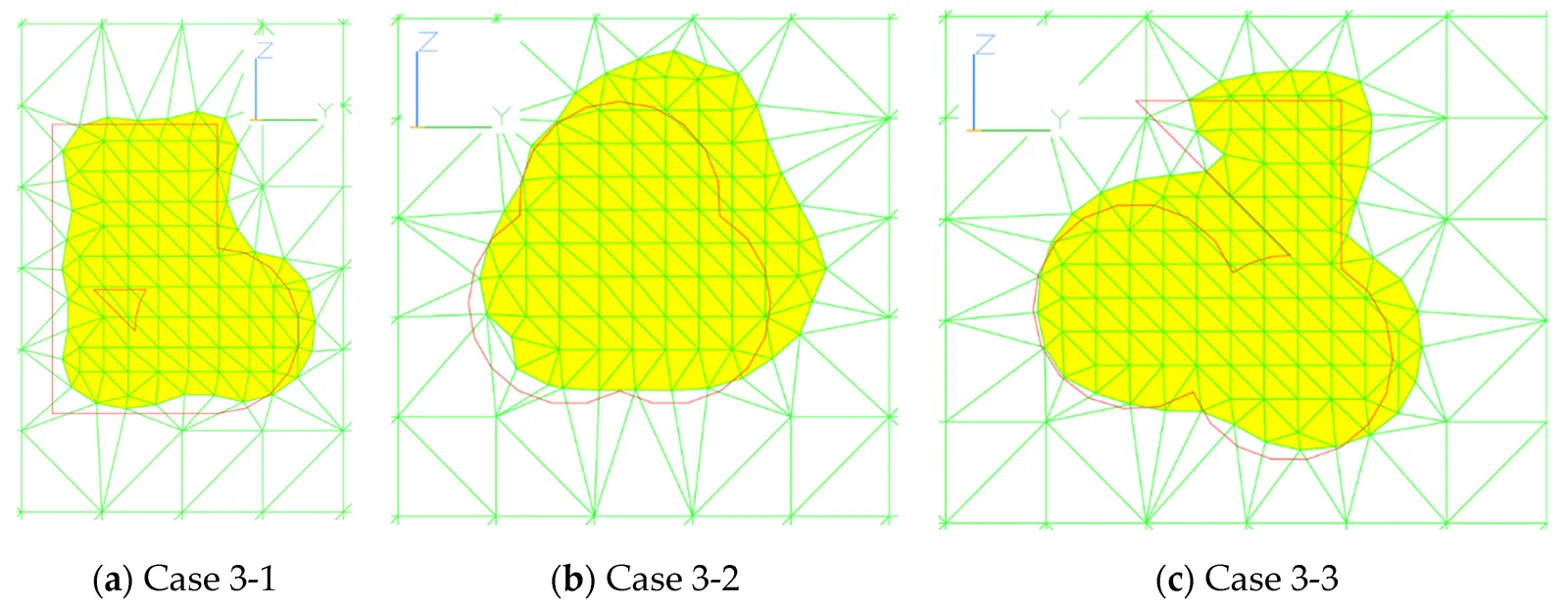
Updated mesh results of Case 3.

**Figure 20:**
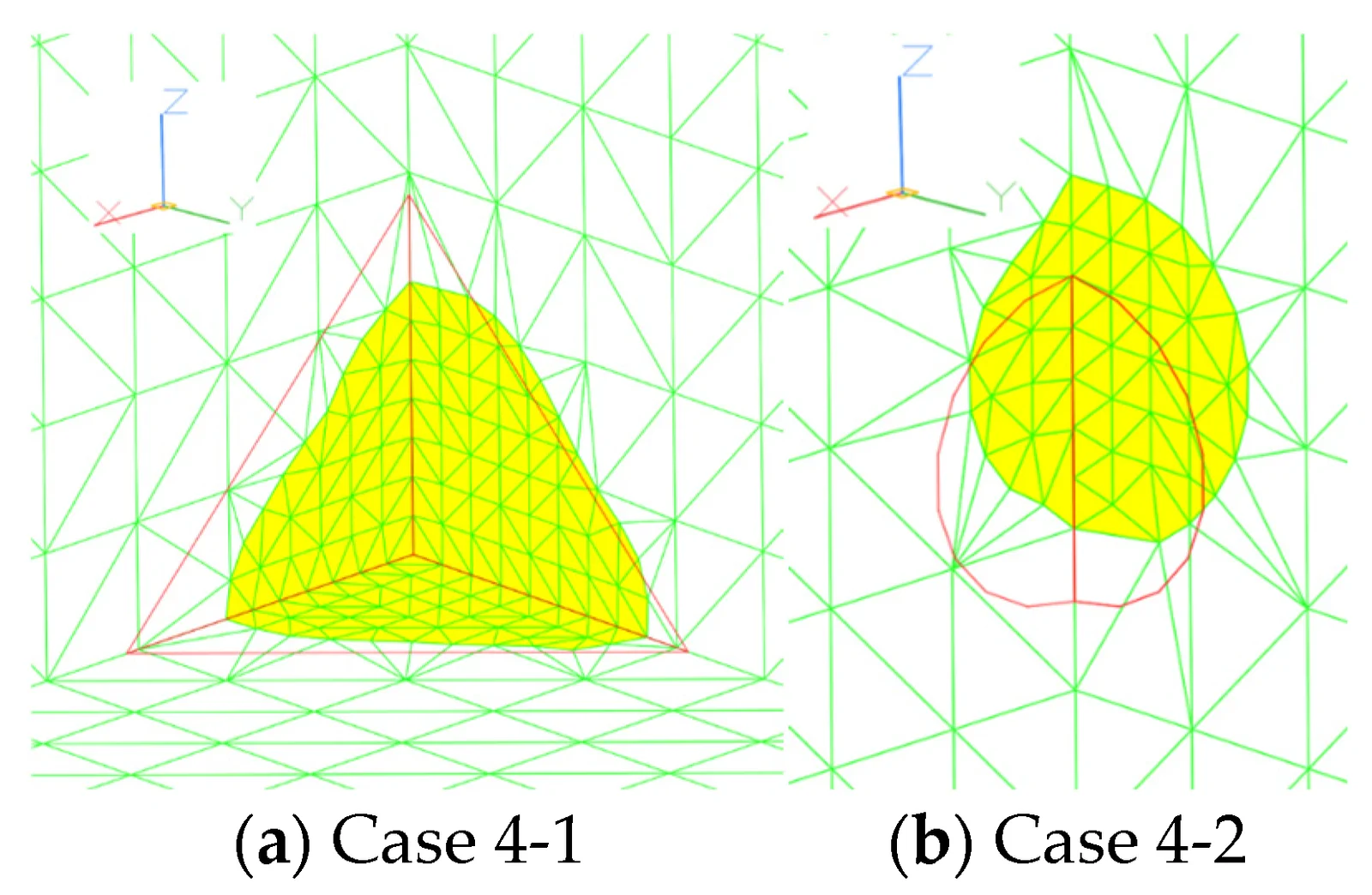
Updated mesh results of Case 4.

Figure 17, Figure 18, Figure 19 and Figure 20 show the detailed updated results for each case, with the red outlines indicating the manually updated results and the yellow regions indicating the PCD-updated results. The Intersection over Union (IoU), calculated using Equation (8), was used to evaluate the geometric quality of the model generated using the PCD-based updating method, as shown in Table 3.

(8) $$\begin{array}{cc} IoU & =\frac{A^{I}}{A^{U}}\\ A^{I} & =A^{manual}\cap A^{PCD}\\ A^{U} & =A^{manual}\cup A^{PCD} \end{array}$$

In Equation (8), the IoU ranges from 0 to 1, where a larger value indicates a closer match between the target and reference shapes. *A*^manual^ is the area enclosed by the outer edge lines of the anomaly-region meshes in the manually updated model, whereas *A*^PCD^ is the area enclosed by the outer edge lines of the anomaly-region meshes generated by the PCD-based updating method presented in this paper.

Table 3 reports IoU values ranging from 0.44 to 0.87, including the individual plate results for the cross-surface cases. The geometric agreement therefore varies substantially among the evaluated regions. Figure 17, Figure 18, Figure 19 and Figure 20 show that the PCD-updated model provides an approximate representation of the anomaly regions, but does not consistently preserve sharp corners, narrow boundary concavities, and small holes.

**Figure 21:**
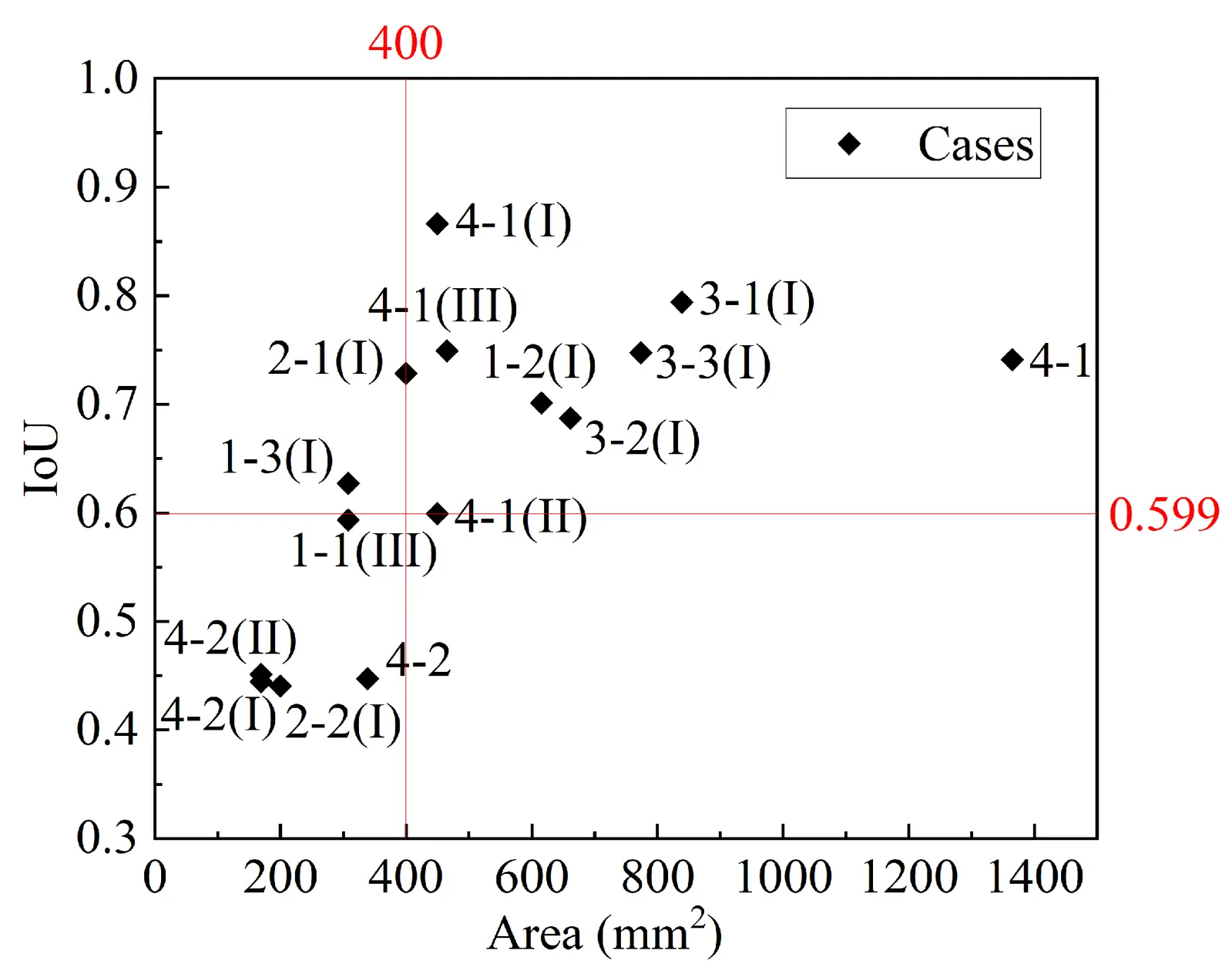
Relationship between anomaly-region area and updating accuracy.

The relationship between the designed anomaly-region area and the corresponding IoU values is presented in Figure 21. Within the present dataset, larger anomaly regions generally exhibit higher IoU values. For designed areas exceeding approximately 400 mm^2^, the IoU values are generally around 0.6 or higher.

**Figure 22:**
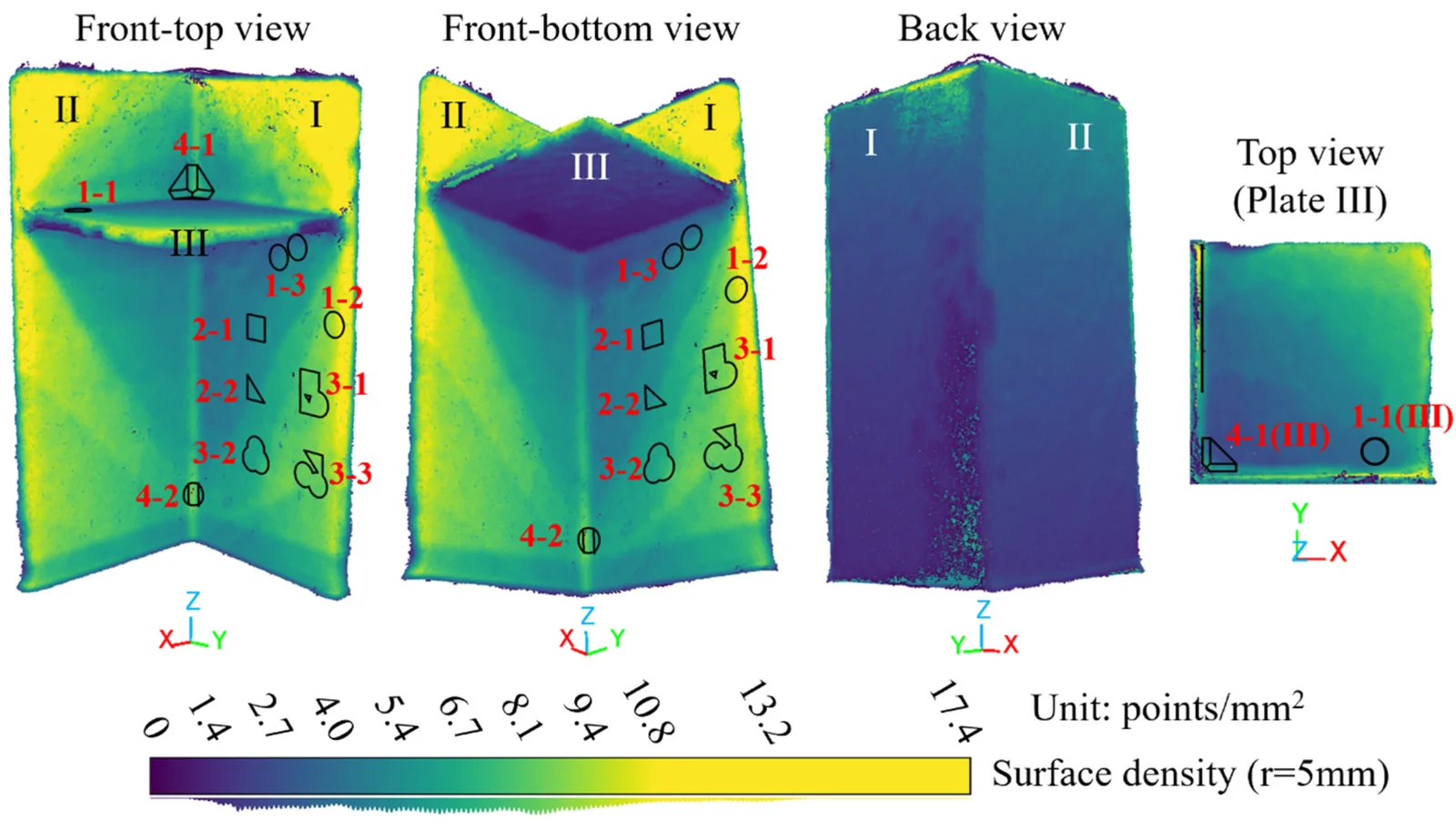
Surface density of the point-cloud data.

Figure 22 presents the surface-density distribution of the point cloud calculated using a neighborhood radius of 5 mm, where the surface density is computed according to Equation (9).

(9) $$\mathrm{Surface}\;\mathrm{Density}=\frac{N_{pn}}{\pi {r_{pn}}^{2}}$$

*N_pn_* denotes the number of central point neighbors inside a sphere of radius *r_pn_*.

The results indicate no obvious correlation between the geometric reconstruction accuracy of the anomaly regions and the local point-cloud surface density. Instead, the influence of point-cloud density is primarily indirect: spatial variations in surface density, even within the same surface, can introduce a bias in the registration result, causing the point cloud to deviate from its expected correct position, where each mesh face should lie on the mid-plane between two surface point clouds of the plate. This bias tends to favor registration solutions in which portions of the point cloud with higher surface density are aligned more closely with the target mesh surface, causing all extracted anomaly regions to be systematically shifted in the same direction relative to the ground truth. Consequently, the geometric reconstruction accuracy is reduced for small anomaly regions, such as Cases 1-1 and 2-2, because even a small registration error results in a relatively large decrease in IoU. Similarly, the anomaly regions extending across multiple surfaces, such as Cases 4-1 and 4-2, are also more susceptible to three-dimensional registration error, making it difficult to maintain high geometric accuracy.

The quality of point cloud and texture mapping also play a more critical role. Lower updating accuracy is often observed in regions with poorer point-cloud quality, as in Cases 1-1 and 1-3 near the plate edge and Case 4-2 that exhibit noticeable texture mapping errors caused by motion blur.

Case 2-2 has an IoU of 0.44 and a designed area of 200 mm^2^, combining a small region size with two acute corners. Its low overlap may reflect both discrepancies in the reconstructed boundary shape and localization errors. Boundary extraction, simplification, smoothing, and resampling may alter fine geometric features during processing. For comparison, Case 2-1 achieves an IoU of 0.73, although its right-angle corners are not fully reproduced. Thus, a relatively high area overlap does not necessarily imply accurate reconstruction of local boundary details.

Case 4-2 represents an anomaly region spanning two adjacent plate surfaces at their intersection and has an overall IoU of 0.45. Each portion has a designed area of 169.3 mm^2^, making its overlap potentially sensitive to localization errors. Reconstruction requires separate boundary extraction and projection on each plate, followed by boundary alignment along the common joint line. Registration and projection errors, together with the observed texture-mapping error, may affect the reconstructed geometry. In contrast, Case 4-1 achieves an overall IoU of 0.74, indicating that reconstruction accuracy is not uniformly low for regions across multiple surfaces. Because the two cases also differ in area, shape, and location, their comparison does not isolate the effect of cross-surface reconstruction. Texture-matching errors may have contributed substantially to the relatively low accuracy in Case 4-2.

The low-IoU cases reveal limitations in the localization of anomaly regions and the preservation of fine geometric features. Future research directions addressing these limitations are outlined in Section 4.

### 3.4. FEA Performance of the Updated Model

A thickness reduction was applied to each of the ten anomaly regions in the two models to simulate corrosion damage on steel structures. According to ISO 9223 [33] and the long-term corrosion model specified in ISO 9224 [34], the predicted corrosion depth of carbon steel after a 100-year service life ranges from approximately 0.02 mm to 13 mm, depending on the atmospheric corrosivity category. Since the objective of this study is to evaluate the FEA applicability of the proposed model updating framework rather than to predict actual corrosion, a severe deterioration scenario was considered. Given that the steel plates in the specimen are only 9 mm thick, excessively large thickness reductions would lead to unrealistic remaining cross-sections and numerical instability or premature local failure, making meaningful comparison between the manually updated (reference) and PCD-updated (target) models difficult. Therefore, based on preliminary trial analyses, a corrosion depth of 6 mm was selected as the maximum thickness reduction that maintained stable structural behavior while producing sufficiently pronounced changes in the structural response. In the global analysis, the prescribed damage was introduced into all anomaly regions. In contrast, in the local analysis, the prescribed damage was introduced only into the target anomaly region to isolate its influence by eliminating interactions with the other damaged regions. The elastoplastic analysis under the same loading and boundary conditions is applied on reference and target models, as shown in Figure 15b.

#### 3.4.1. Global Response Evaluation

**Figure 23:**
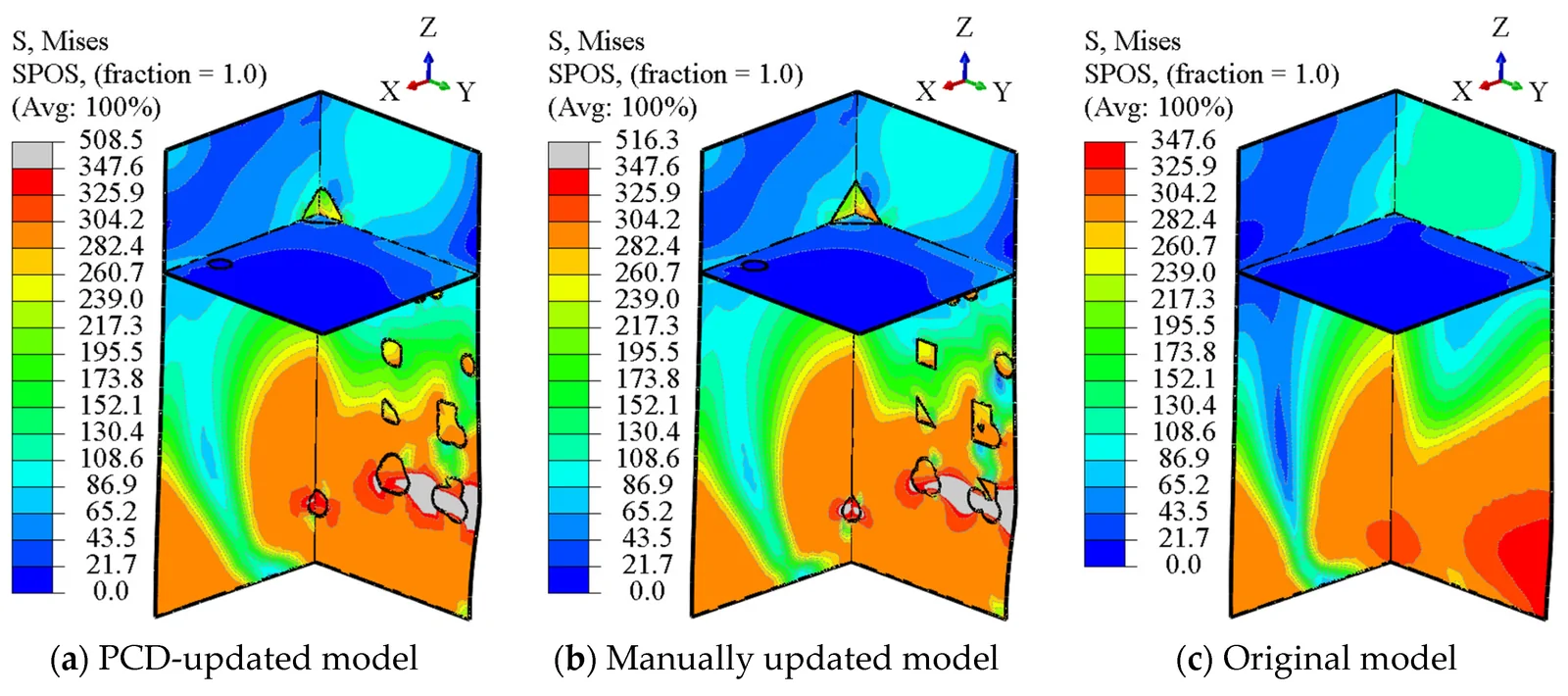
Global Mises stress distribution (MPa).

**Figure 24:**
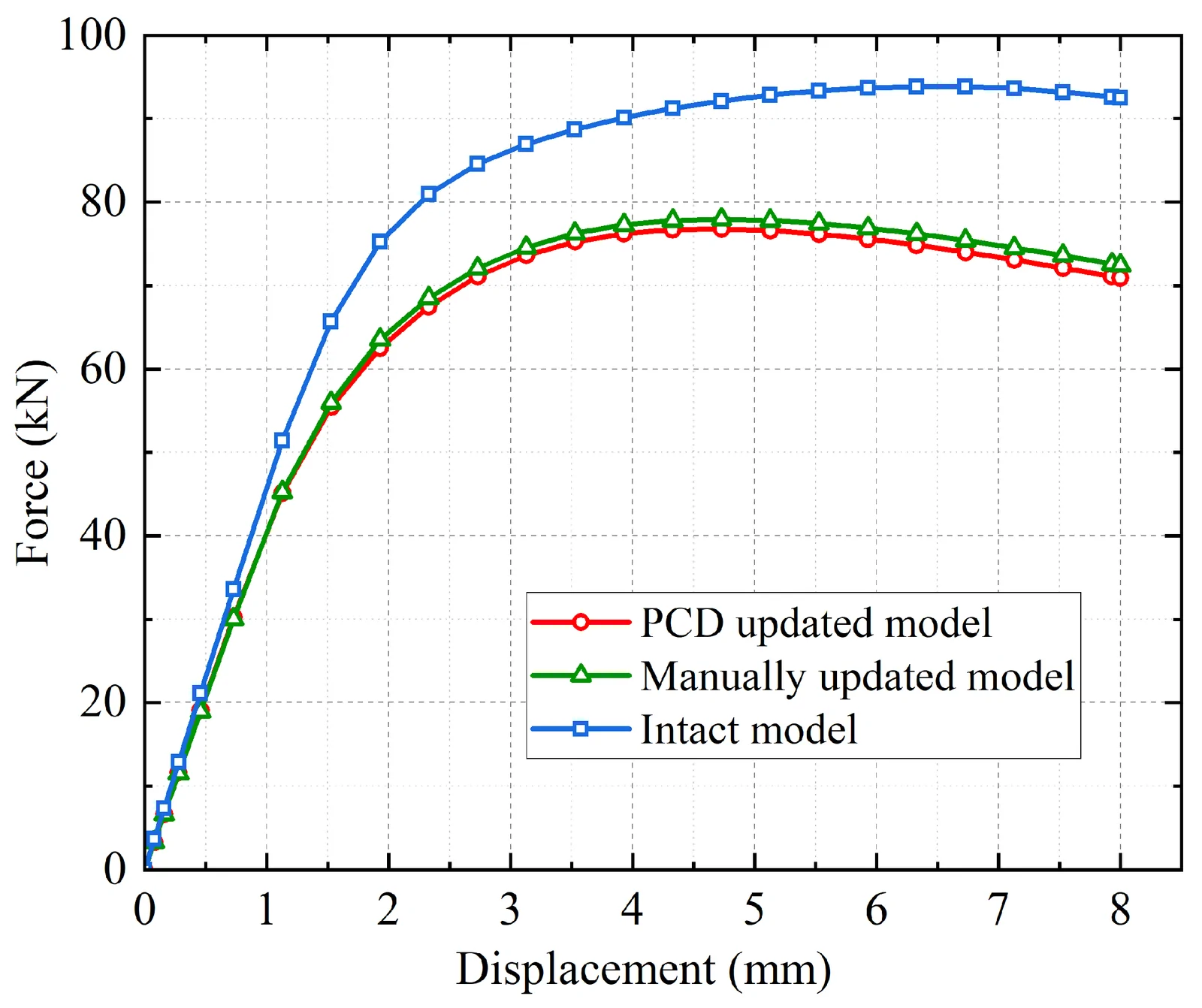
Load–displacement curves at the loading point for the original, PCD-updated, and manually updated FE models.

Figure 23 shows the global von Mises stress distribution contours for the three models of the specimen under the same displacement loading of 8 mm. To unify the intervals of the contour line, the stress display range was limited from 0 to 347.6 MPa, which is the maximum stress in the original model. The stress over limitation is masked by gray color. The surface directions are shown in Figure 15b. Figure 24 shows the load–displacement curves at the loading point for the three models during the loading process. The von Mises stress distributions of the manually updated and PCD-based updated models are generally consistent. Compared with the original model, both updated models exhibit a noticeable distortion of stress contours near the damaged region, which results from the stress concentration induced by the local thickness reduction. The deformation patterns of the contours are essentially consistent between the two models. A more detailed quantitative comparison is presented in the following sections.

The load–displacement curves also clearly reflect the reduction in load capacity due to damage, with the curves of the damaged models showing a significant decrease in the peak load. The ultimate load-bearing capacity was defined as the maximum load immediately before the descending branch of the load–displacement curve. The corresponding displacement was also recorded to characterize the deformation required to reach the ultimate load-bearing capacity. The ultimate load-bearing capacities and corresponding displacements of the three models are listed in Table 4. Compared with the original FE model, the manually updated and PCD-updated models predict reductions of 17.0% and 18.2%, respectively, in the ultimate load-bearing capacity. Both models predict an identical 25.3% reduction in the displacement corresponding to the ultimate load. The predicted reductions in ultimate load-bearing capacity differ by only 1.2 percentage points between the two updated models, indicating good agreement between their corresponding results.

#### 3.4.2. Local Response Evaluation

**Figure 25:**
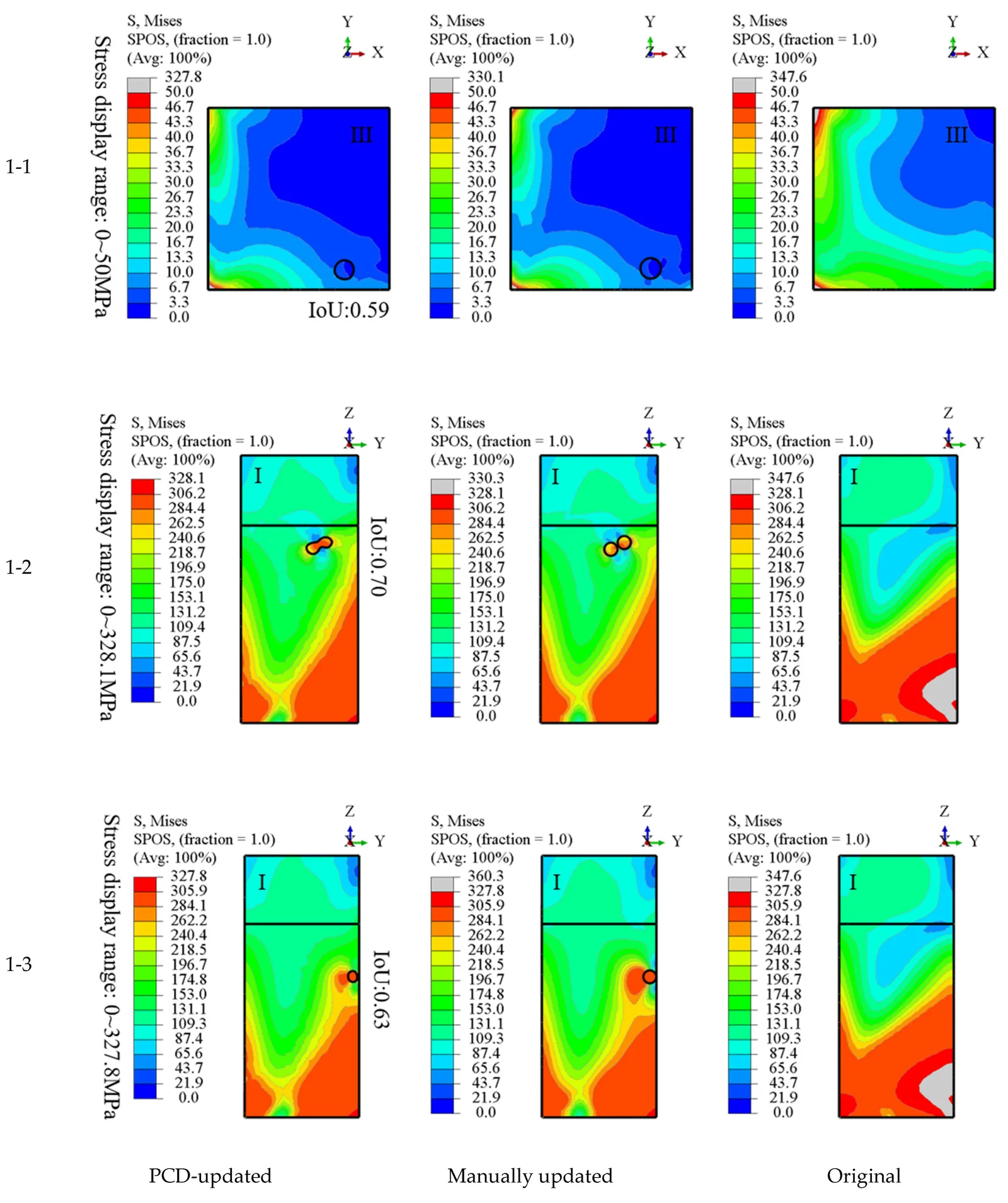
Local stress distributions in Case 1 (unit: MPa).

**Figure 26:**
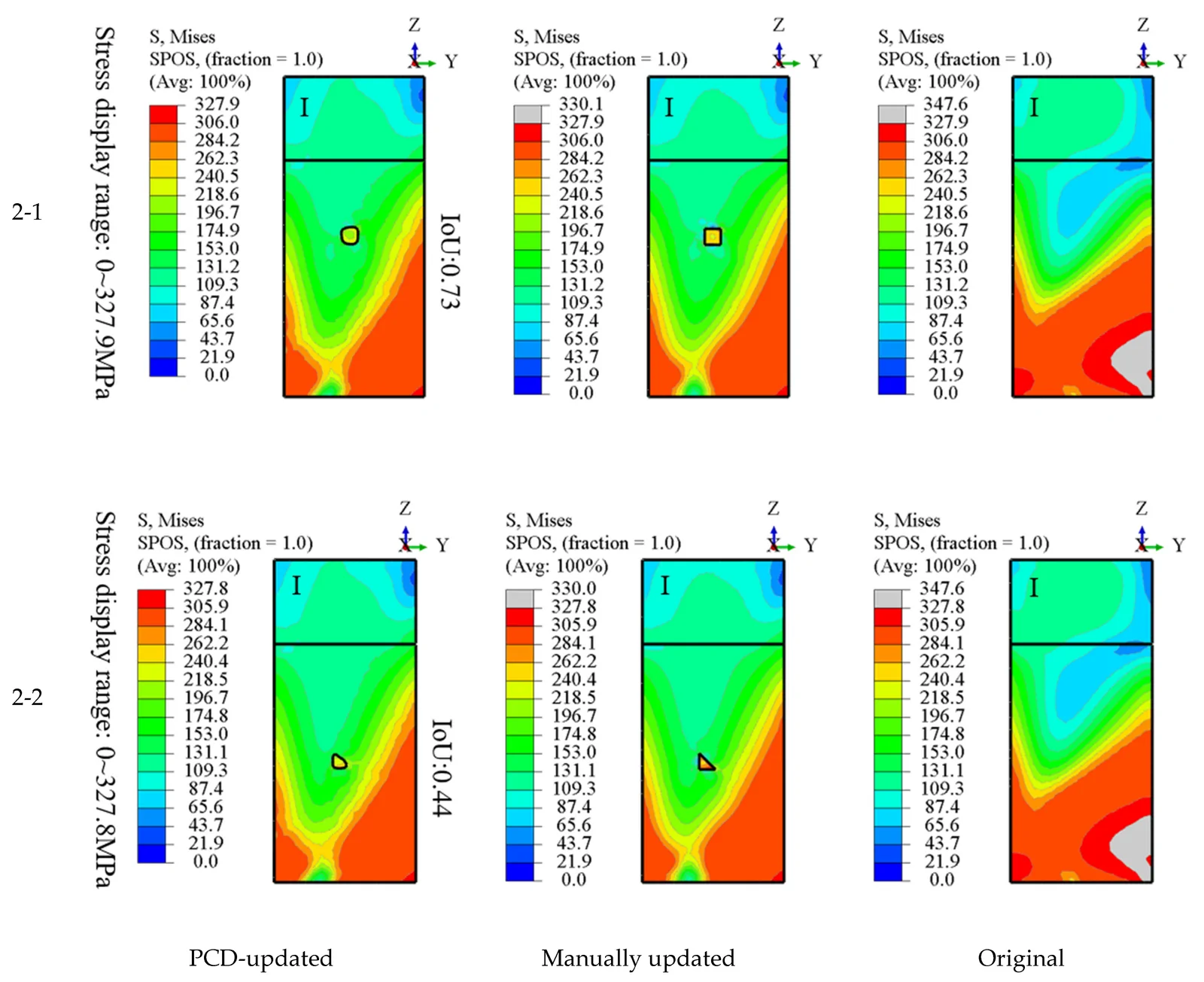
Local stress distributions in Case 2 (unit: MPa).

**Figure 27:**
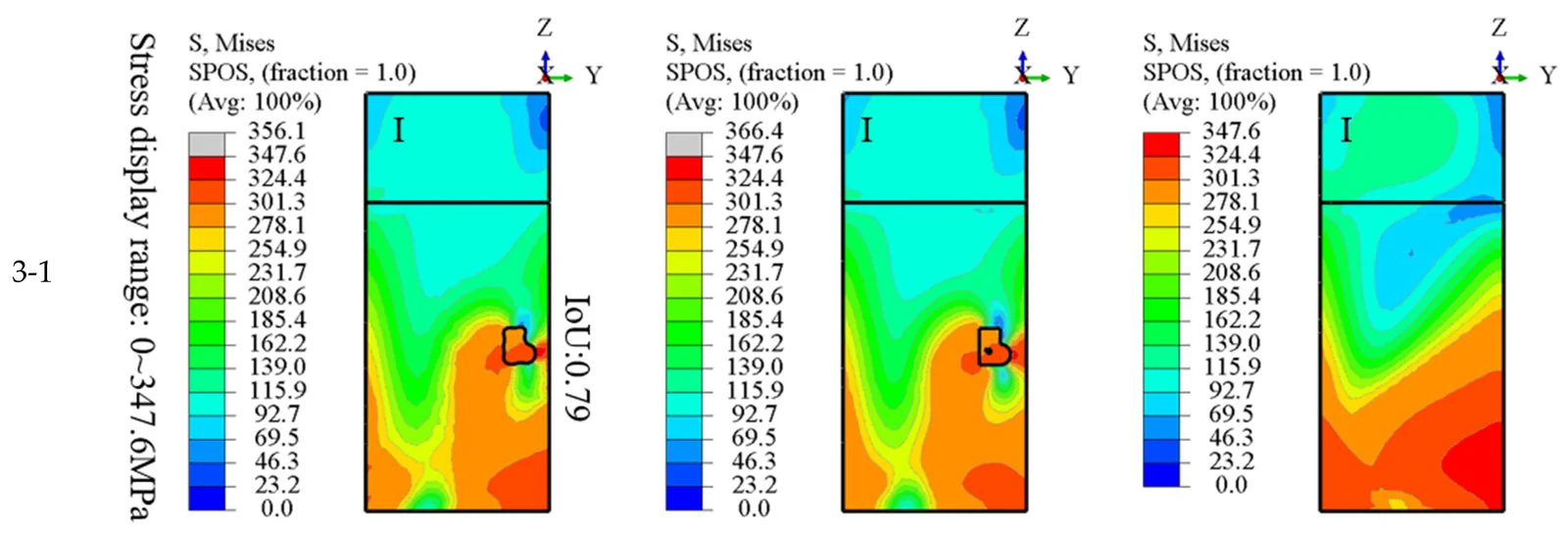

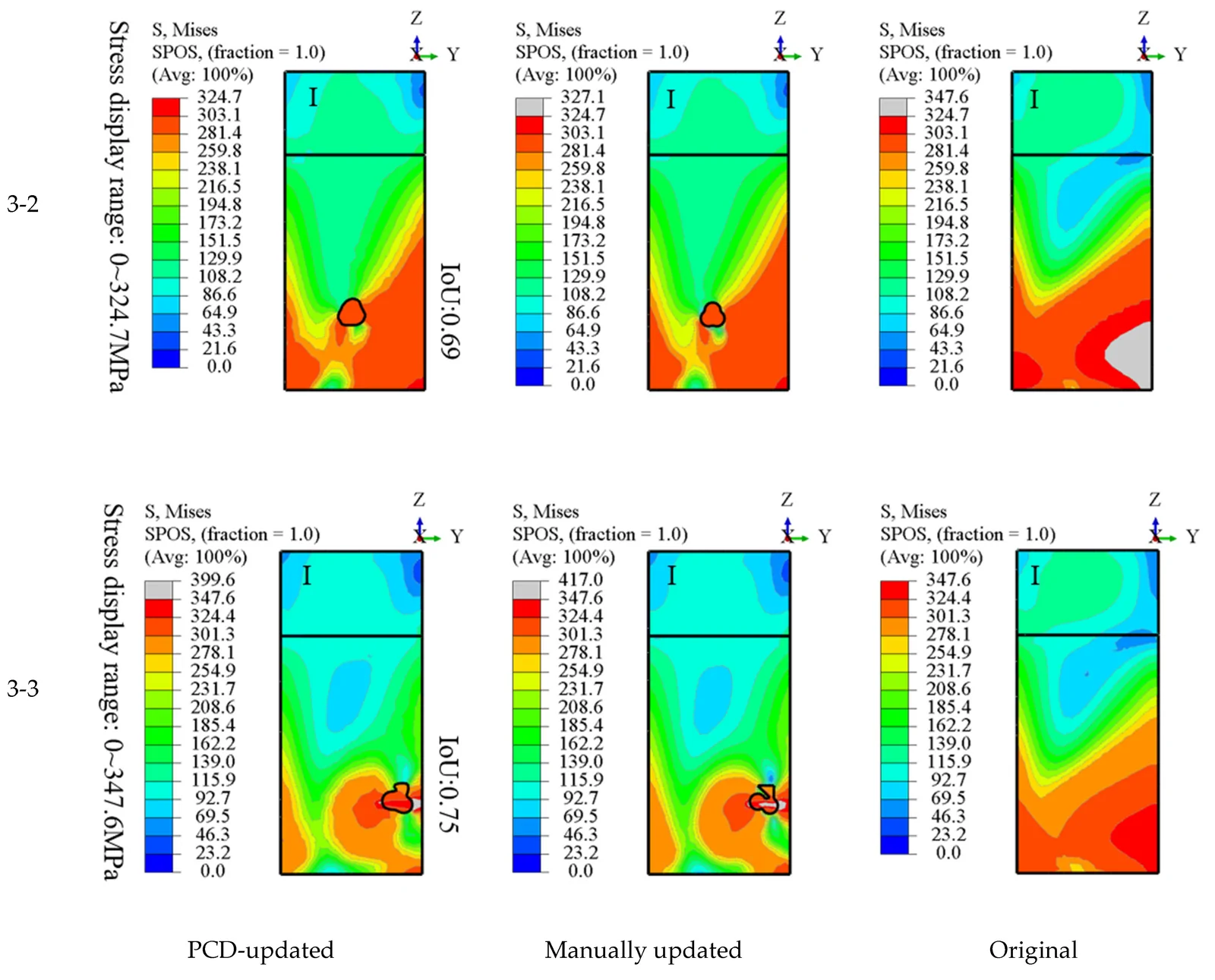
Local stress distributions in Case 3 (unit: MPa).

**Figure 28:**
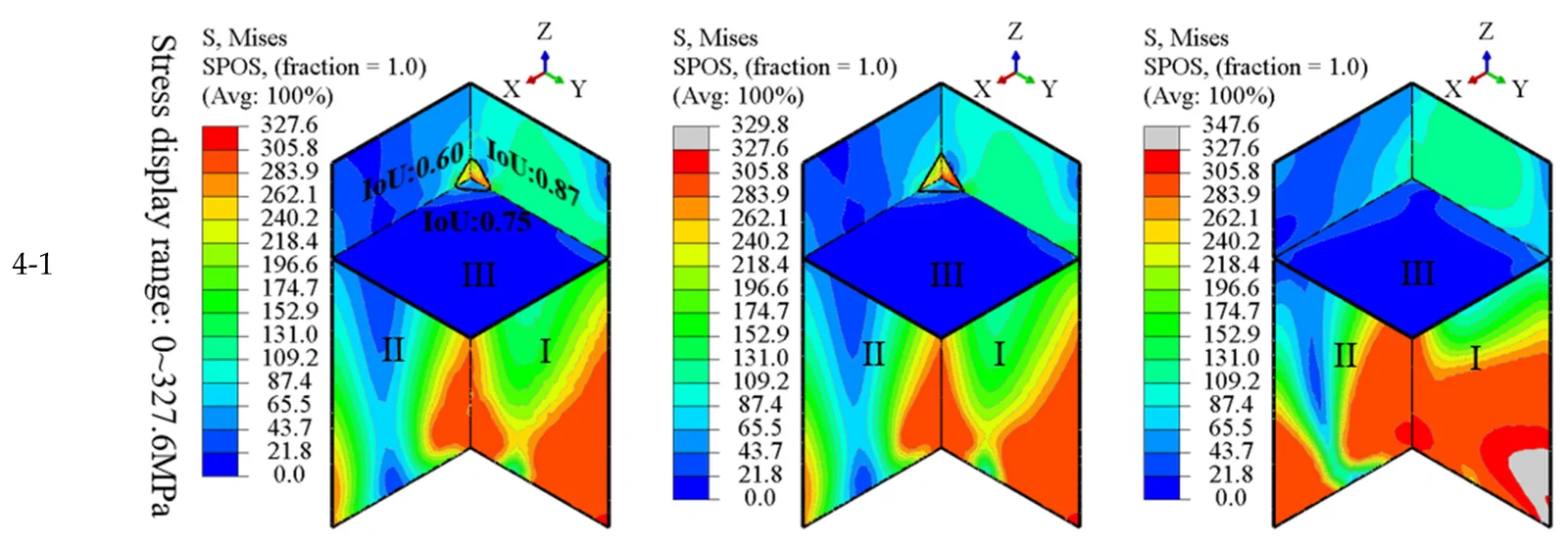

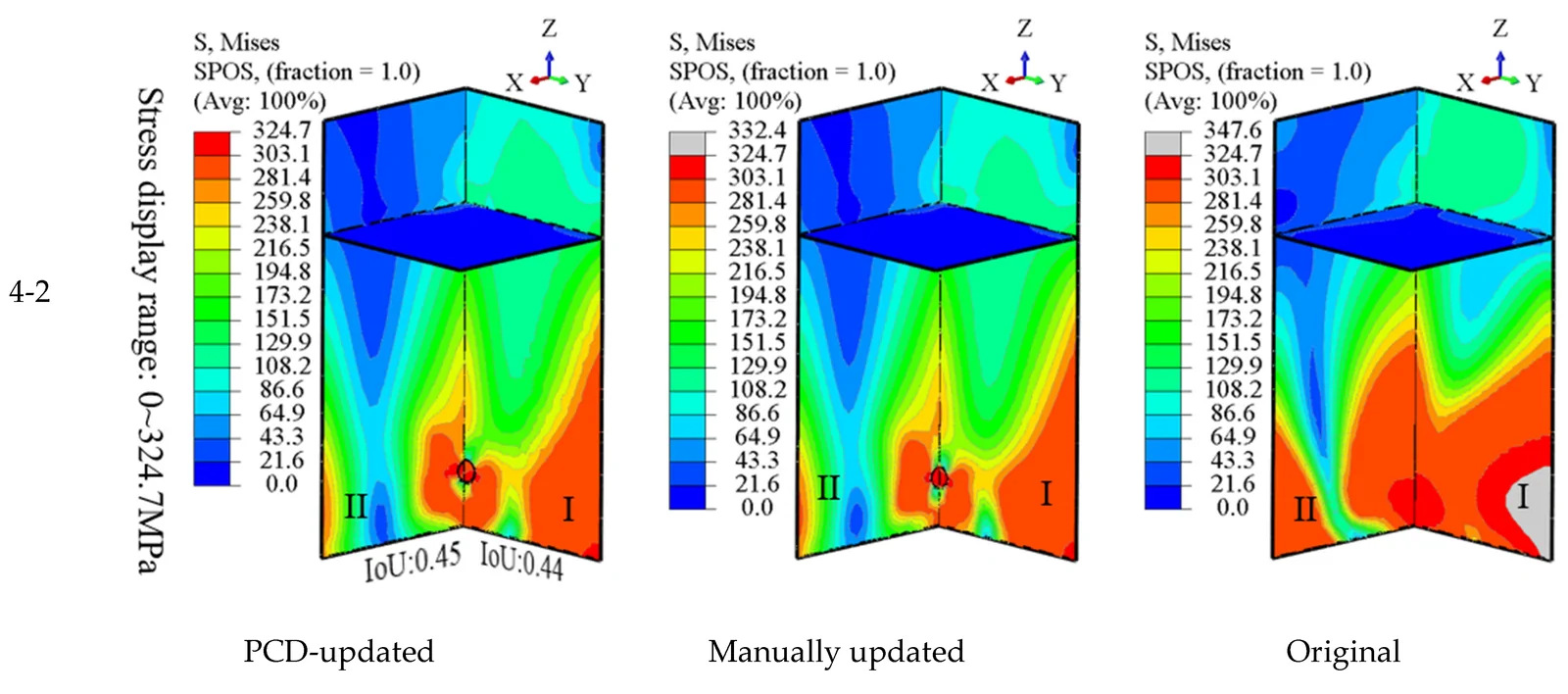
Local stress distributions in Case 4 (unit: MPa).

To verify whether the two updating methods yield consistent effects on the local stress distribution in the damaged regions, Figure 25, Figure 26, Figure 27 and Figure 28 illustrate the impact of the damage on the local stress distributions across all four cases under a displacement load of 3 mm. The thickness-reduction damage was introduced only into the target anomaly region to exclude interactions with nearby damage. To clearly illustrate the stress changes around the damaged regions, all visualizations are presented with the plate containing the damaged region as the primary view. To facilitate comparison of the stress distributions among the three models, the same stress display range and consistent contour intervals were used for three models in each case. The maximum stress was first obtained for each model, and the lowest of the three maximum stress values was used as the upper limit for each case, while the lower limit of displayed stress was uniformly set to 0 MPa. Stress values exceeding the upper limit were masked in gray. For Case 1-1, however, the stresses and their variations in the transverse stiffener were relatively small compared with those in the overall structure. Therefore, the stress display range for all three models was limited to 0–50 MPa to better highlight the local stress variations. The models updated using the proposed method and those manually updated according to the design drawings successfully capture the stress concentration induced by the damage, demonstrating consistency in the analysis results of the two models.

For the damaged regions with smoothly varying boundaries (Case 1), the stress distributions around the damaged regions generally show good agreement between the two models. However, for the damaged regions with abrupt curvature changes at the boundary (Cases 2 and 3), the PCD-updated models tend to underestimate the stress concentration typically observed at sharp tips in the manually updated model. Additional discrepancies are associated with incomplete reconstruction of small geometric features. In Case 1-2, the narrow gap between two closely spaced anomaly regions is not accurately reconstructed because of poor surface-texture quality. In Case 3, the small internal hole in Case 3-1 and the boundary concavities in Cases 3-2 and 3-3 are not accurately preserved, particularly the high-aspect-ratio concavity in Case 3-3. These geometric discrepancies may contribute to differences in the magnitude and location of local stress concentrations.

The cross-surface cases 4-1 and 4-2 show qualitative similarities in stress redistribution, but discrepancies remain in the magnitude and location of local stress concentrations.

**Figure 29:**
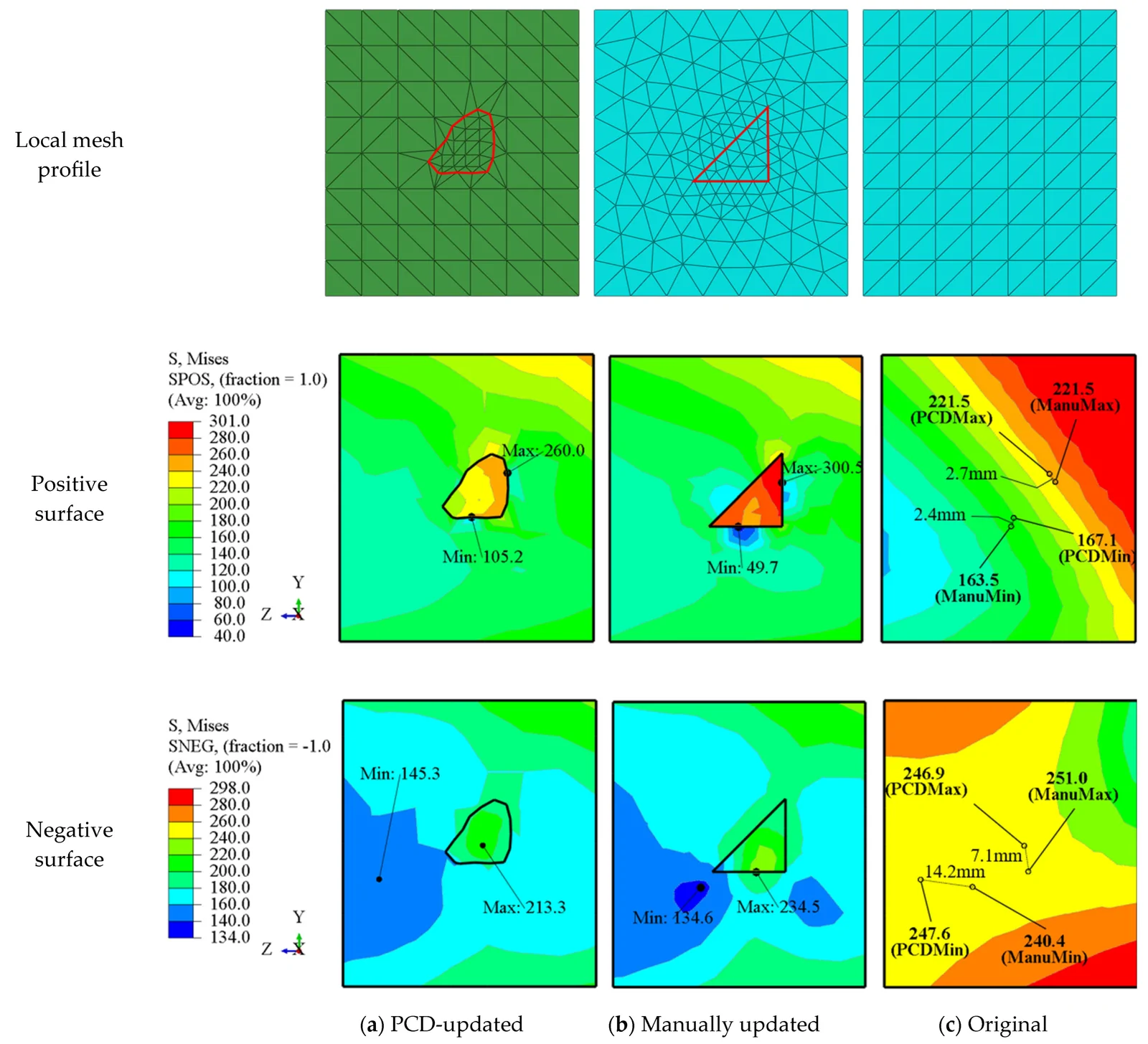
Stress distributions around damaged regions of Case 2-2 (surface direction is illustrated in Figure 15b).

Moreover, in the damaged regions with lower geometric accuracy in the PCD-updated model (IoU_2-2_:0.44; IoU_4-2_:0.45), both the magnitude and extent of stress concentration tend to be significantly underestimated, and the locations of the local peak stress also shift. More detailed analysis was conducted for Case 2-2, which exhibits the largest geometric discrepancy among all cases. As shown in Figure 29, the damage introduces stress concentrations within the anomaly region, increasing the local stress while simultaneously relieving part of the stress in the surrounding region. The pattern of local stress redistribution captured by PCD-updated and manually updated models is similar. However, the PCD-updated model underestimates the magnitude and shifts the location of the local stress concentration which may partly reflect the incomplete reconstruction of the sharp tip. Within the damage-affected region, the absolute differences between the two updated models in the percentage changes of local stress extrema, relative to the corresponding original-model stresses, ranged from 2.7 to 32.6 percentage points. The location shift between corresponding extrema ranged from 2.4 to 14.2 mm. The detailed stress results are summarized in Table 5.

#### 3.4.3. Summary

Based on the present numerical comparison, the PCD-updated model shows good agreement with the manually updated reference model in the global load response and qualitative agreement in the stress concentration patterns near the anomaly regions. However, quantitative discrepancies remain in the magnitude and location of local stress concentrations. Differences in reconstructed geometry and mesh topology may both contribute to these discrepancies. This is particularly relevant near sharp boundaries, where the stress distribution is sensitive to both mesh and geometry. The individual effects of these factors have not been isolated. The global load response therefore provides a more robust indicator of consistency than local stress extrema. Further sensitivity studies are needed to assess the reliability of quantitative local stress predictions.

## 4. Conclusions

In this study, a modular PCD-based mesh-model updating framework was proposed to incorporate surface anomaly regions into existing shell-element FE models of thin-walled steel structures through the joint use of color and geometric information. The framework adapts and integrates established algorithms with full consideration of the characteristics of thin-walled structures, their FE models, and point-cloud data, thereby achieving methodological innovation through their integration.

A proof-of-concept evaluation of the proposed framework was conducted by comparing the PCD-updated model with a manually updated reference FE model in terms of geometric accuracy and consistency of FE responses. The evaluation used point-cloud data acquired with a single scanner from one steel angle member specimen with ten predefined, high-contrast anomaly regions. The main conclusions are summarized as follows:

(1) Under the controlled conditions in this study, the results demonstrate the feasibility of incorporating surface anomaly regions into existing shell-element FE models while accounting for the offset between the measured surface and the mid-plane mesh, as well as maintaining mesh continuity when anomaly regions extend across plate joints.

(2) The geometric evaluation indicates that the framework can represent the approximate spatial extent of the investigated anomaly regions, with IoU values ranging from 0.44 to 0.87, but does not consistently reproduce all local boundary details. Sharp corners, narrow concavities, and small holes are hard to preserve.

(3) The FE comparisons demonstrate the potential of the proposed approach for preliminary FE-based assessment, with stronger agreement between the updated and manually constructed reference models in the global load response than in quantitative local stress predictions. The predicted reductions in ultimate load-bearing capacity differed by approximately 1.2 percentage points between the two models. The local stress redistribution showed qualitative agreement, although quantitative discrepancies remained. These discrepancies may arise from differences in the reconstructed geometry and mesh topology, but the individual contributions of these factors have not yet been isolated.

The numerical comparison does not constitute experimental validation of the response of a physically damaged structure. Artificial color anomalies do not reproduce the roughness variations, gradual color transitions, or irregular material loss associated with actual corrosion. Applicability to actual corrosion and other structural configurations requires further investigation. Validation against mechanical testing on steel specimens with actual corrosion is still needed. Methodological improvements should focus on enhancing registration accuracy and developing more robust methods for representing corrosion boundaries. Further studies should assess the robustness and sensitivity of individual modules, particularly the effects of boundary identification resolution, algorithm parameters, and ambient light as well as point-cloud and mesh-model quality. Error propagation throughout the framework and computational efficiency should also be investigated.

## Figures and Tables

**Table 1:** Principal processing parameters used in the proposed framework.

Processing Step	Parameter	Value or Rule
Alpha-shape search	Search bounds	0.01~3
	Maximum number of iterations	100
	Retained point ratio of down-sampling	40%
	Number of sampling trials	3
	Final *α* value	Median of the three returned values
RDP simplification	Tolerance, *ε*	0.2 mm
B-spline smoothing	Degree, *p*	3
	Smoothing factor, *s*	10
	Periodic fitting	Enabled
Feature point arrangement	Point-spacing threshold, *d_T_*	D/3 *

* *D* is the mean distance from a vertex to its two nearest neighboring vertices.

**Table 2:** Mesh characteristics of the compared finite element models.

Model	PCD-Updated	Manually Updated	Original
Number of nodes	2416	4212	1841
Number of elements	4678	8266	3528
Element type	S3	S3	S3

**Table 3:** Calculation results for the Intersection over Union.

Case Number	Plate Number	Designed Area (mm^2^)	Intersection Area (mm^2^)	Union Area (mm^2^)	IoU
1-1	III	307.83	214.57	361.58	0.59
1-2	I	615.65	468.83	668.58	0.70
1-3	I	307.83	193.09	307.83	0.63
2-1	I	400	331.97	455.84	0.73
2-2	I	200	126.44	286.96	0.44
3-1	I	839.13	741.93	934.14	0.79
3-2	I	661.52	613.50	892.93	0.69
3-3	I	773.75	729.23	975.36	0.75
4-1	I	449.89	415.34	479.34	0.87
	II	449.89	269.58	449.96	0.60
	III	465.14	348.48	465.14	0.75
	I + II + III	1364.92	1033.40	1394.43	0.74
4-2	I	169.3	132.95	299.04	0.44
	II	169.3	89.20	197.63	0.45
	I + II	338.6	222.15	496.67	0.45

**Table 4:** Comparison of ultimate load-bearing capacities.

	PCD-Updated	Manually Updated	Original
Maximum load (N)	76,799.2	77,944.4	93,895.3
Reduction from original (%)	18.21	16.99	-
Displacement in maximum load (mm)	4.73	4.73	6.33
Reduction from original (%)	25.28	25.28	-

**Table 5:** Comparison of stress extremes around damaged region of Case 2-2.

Surface	Mises Stress	PCD-Updated Model			Manually Updated Model			Differences Between Two Updating Methods (Percentage Points)
		Updated (MPa)	Ref. (MPa)	Change (%)	Updated (MPa)	Ref. (MPa)	Change (%)	
Positive	Max	260	221.5	+17.4	300.5	221.5	+35.7	−18.3
	Min	105.2	167.1	−37.0	49.7	163.5	−69.6	32.6
Negative	Max	213.3	246.9	−13.6	234.5	251	−6.6	−7.0
	Min	145.3	247.6	−41.3	134.6	240.4	−44.0	2.7

## Data Availability

The datasets presented in this article are not readily available due to technical limitations. Requests to access the datasets should be directed to co-author, Mayuko Nishio (Email: nishio@kz.tsukuba.ac.jp).

## Abbreviations

The following abbreviations are used in this manuscript:

2D	Two-dimensional
3D	Three-dimensional
CAD	Computer-aided design
CAE	Computer-aided engineering
CDT	Constrained Delaunay triangulation
CIELab	CIE L*a*b* color space
CV	Computer vision
DLT	Direct linear transformation
DOF	Degrees of freedom
FE	Finite element
FEA	Finite element analysis
FOREST	Fusion-Oriented REsearch for disruptive Science and Technology
HSV	Hue, saturation, and value
ICP	Iterative closest point
IoU	Intersection over Union
ISO	International Organization for Standardization
LiDAR	Light detection and ranging
OR	Overlap rate
PCA	Principal component analysis
PCD	Point-cloud data
RC	Reinforced concrete
RGB	Red, green, and blue
RGB-D	Red, green, blue, and depth
SVD	Singular value decomposition
UAV	Unmanned aerial vehicle
YOLOv5	You Only Look Once version 5
